# Combinative effects of akarkara root-derived metabolites on anti-inflammatory and anti-alzheimer key enzymes: integrating bioassay-guided fractionation, GC-MS analysis, and *in silico* studies

**DOI:** 10.1186/s12906-023-04210-6

**Published:** 2023-11-17

**Authors:** Rana M. Ibrahim, Passent M. Abdel-Baki, Ghada F. Elmasry, Ahmed A. El-Rashedy, Nariman E. Mahdy

**Affiliations:** 1 Pharmacognosy Department, Faculty of Pharmacy, Cairo University, Kasr El-Ainy Street, Cairo, 11562 Egypt; 2 Department of Pharmaceutical Chemistry, Faculty of Pharmacy, Cairo University, Kasr El-Aini Street, Cairo, 11562 Egypt; 3 Natural and Microbial Products Department, National Research Center (NRC), Dokki, Giza 12622 Egypt

**Keywords:** Akarkara root, GC-MS, Anti-inflammatory, Anticholinergic, Molecular docking, ADME

## Abstract

**Background:**

*Anacyclus pyrethrum* L. (Akarkara root), a valuable Ayurvedic remedy, is reported to exhibit various pharmacological activities. Akarkara root was subjected to bioassay-guided fractionation, to isolate its active constituents and discover their potential bioactivities, followed by computational analysis.

**Methods:**

The methanol extract and its fractions, methylene chloride, and butanol, were assessed for their antioxidant, anti-inflammatory, and anticholinergic potentials. The antioxidant activity was determined using DPPH, ABTS, FRAP, and ORAC assays. The in vitro anticholinergic effect was evaluated via acetyl- and butyryl-cholinesterase inhibition, while anti-inflammatory effect weas determined using COX-2 and 5-LOX inhibitory assays. The methylene chloride fraction was subjected to GC/MS analysis and chromatographic fractionation to isolate its major compounds. The inhibitory effect on iNOS and various inflammatory mediators in LPS-activated RAW 264.7 macrophages was investigated. *In silico* computational analyses (molecular docking, ADME, BBB permeability prediction, and molecular dynamics) were performed.

**Results:**

Forty-one compounds were identified and quantified and the major compounds, namely, oleamide (**A1**), stigmasterol (**A2**), 2E,4E-deca-2,4-dienoic acid 2-phenylethyl amide (**A3**), and pellitorine (**A4**) were isolated from the methylene chloride fraction, the most active in all assays. All compounds showed significant in vitro antioxidant, anticholinergic and anti-inflammatory effects. They inhibited the secretion of pro-inflammatory cytokines (TNF-*α*, IL-1*β*, and IL-6) in activated RAW macrophages. The isolated compounds showed good fitting in the active sites of acetylcholinesterase and COX-2 with high docking scores. The ADME study revealed proper pharmacokinetics and drug likeness properties for the isolated compounds. The isolated compounds demonstrated high ability to cross the BBB and penetrate the CNS with values ranging from 1.596 to -1.651 in comparison with Donepezil (-1.464). Molecular dynamics simulation revealed stable conformations and binding patterns of the isolated compounds with the active sites of COX-2 and acetyl cholinesterase.

**Conclusions:**

Ultimately, our results specify Akarkara compounds as promising candidates for the treatment of inflammatory and neurodegenerative diseases.

**Supplementary Information:**

The online version contains supplementary material available at 10.1186/s12906-023-04210-6.

## Background

Non-communicable diseases are becoming more common throughout the world and tend to be of long duration. They are the result of a combination of genetic, physiological, environmental, and behavioral factors like Alzheimer’s disease (AD), cancers, diabetes, and chronic cardiovascular diseases. AD is a progressive, age-related neurodegenerative condition that affects the elderly. Memory loss, a deterioration in language abilities, and other cognitive deficits are characteristics of AD. Around 50 million people are estimated to have dementia according to the World Health Organization (WHO), with AD being the most prevalent form, accounting for 60–70% of all cases. It is concerning because 82 million people are anticipated to experience AD globally by 2030 and 152 million by 2050 [1]. One of the major difficulties facing healthcare systems is the financial impact of dementia in terms of direct medical and social care expenditures. According to The World Alzheimer Report (2015), the expense of caring for AD patients would reach 2 trillion US$ by 2030, undermining global social, and economic advancement besides, overwhelming health and social services, including long-term care systems [2]. Oxidative stress is the main obstacle in the majority of diseases. The imbalance between the generation of free radicals and reactive metabolites starts to destroy vital cells and biomolecules, potentially having an impact on the health of the entire body. An antioxidant defense mechanism is any defensive mechanism that eliminates such free radicals [3]. By synchronizing the destructive activity of free radicals, the antioxidant defense mechanism plays a crucial part in reducing free radicals. They mainly target macromolecules that damage cells [4]. The etiology of AD is significantly influenced by these oxidative stress, inflammation and decreased acetylcholine levels [5, 6]. As a result, treating high levels of oxidative stress prevents the progression of numerous disorders, including Alzheimer’s disease [7]. There are many theories have been proposed for the pathophysiology of AD, such as dysfunction of the cholinergic neurons system, deposits of *β*-amyloid (A*β*) protein, *τ*-protein hyperphosphorylation, and metal dyshomeostasis [8]. According to the cholinergic hypothesis, increasing acetylcholine levels in the brain by blocking cholinesterase enzymes is an effective therapeutic strategy for treating the symptoms of Alzheimer’s disease. This is achieved through inhibiting acetyl- (AChE) and butyryl-cholinesterase (BChE). However, this therapeutic strategy simply provides symptomatic alleviation rather than a drastic cure, failing to stop or considerably slow the disease’s course [9]. Neuroinflammation triggered by inflammatory mediators such as prostaglandins [by cyclooxygenase-2 (COX-2)], leukotrienes [by 5-lipoxygenase (5-LOX)], and nitric oxide [by inducible nitric oxide synthase (iNOS)], as well as pro-inflammatory cytokines [tumor necrosis factor-alpha (TNF-*α*), and interleukins (IL-1*β* and IL-6)] has been shown to play a role in the etiology of AD [10], along with oxidative stress [8]. Free radicals are produced in greater quantities when an inflammatory process begins in the body. Consequently, it is preferable to provide the patient with a suitable antioxidant as an additional treatment for Alzheimer’s and neuroinflammation. Drugs that modify the activity of a particular target may not be sufficient to stop the course of AD due to the complexity of the illness. Therefore, the development of multi-targeted medications that combine various pharmacological actions is more likely to result in effective therapy.

*Anacyclus pyrethrum* L. (Asteraceae), commonly known as Akarkara, is native to Asia and Africa [11]. The roots and leaves of the plant are well documented in Ayurvedic and Unani systems of holistic healing. *A. pyrethrum* is used as a brain tonic in complementary and alternative medicine [12]. In traditional North African and Indian medicine, *A. pyrethrum* roots are used to treat several diseases, including Alzheimer’s disease, diabetes, anabolic, aphrodisiac, reproductive, anti-rheumatic, analgesic, antibacterial, antiviral, carminative, anti-catarrhal, digestion, febrifuge, nervine, vermifuge, and sialagogue [13, 14]. In Indian ayurvedic medicine, it is widely used for the treatment of male infertility [15]. In siddha medicine, it is used for treatment of arthritis [16]. It is widely used in the treatment of epilepsy, seizures and lung infections, as well as its use as an insecticide in ethnomedicines [17]. *A. pyrethrum* roots were reported to exhibit memory-enhancing, anticonvulsant, antioxidant, neuropharmacological, anti-inflammatory, analgesic, wound healing, immunostimulant, antibacterial, androgenic, antiplasmodial and insecticidal activities [13, 18, 19]. Several interesting active constituents were reported in *A. pyrethrum* including pyrethrins, alkylamides, and polysaccharides [19]. Despite the various ethnopharmacological and phytotherapeutic reports regarding its neuropharmacological, anti-inflammatory, and antioxidant activities, nothing was traced about its potential as a multi-targeted AD drug.

There is a significant shift in drug discovery from synthetic moieties to herbal formulations for the treatment of neurological disorders and their scientific validation to cure neurodegenerative disorders. Therefore, this study was conducted to highlight the beneficial effects of *A. pyrethrum* roots as antioxidant, anti-inflammatory, and anticholinergic drug that can be used to prevent or manage the symptoms or halt the progression of neurodegenerative diseases. The methanol extract (ME) of the roots and its fractions [methylene chloride (MCF) and butanol (BF)] were tested for their antioxidant capacities using different methods, such as DPPH (2,2-diphenyl-1-picrylhydrazyl), ABTS (2,2’-azino-di(3-ethylbenzthiazoline-6-sulfonic acid, FRAP (ferric reducing antioxidant power), ORAC (oxygen radical absorbance capacity), and metal chelation assay. Furthermore, the in vitro anticholinergic (AChE and BChE) and anti-inflammatory (COX-2 and LOX) effects of all extracts were evaluated. Furthermore, the effects of Akarkara root extract, its fractions, and isolated compounds on cell viability of RAW264.7 macrophages were assessed by MTT (3-[4,5-dimethylthiazol-2-yl]-2,5 diphenyl tetrazolium bromide) assay. The inhibition of iNOS and pro-inflammatory cytokines (TNF-*α*, IL-1*β*, and IL-6) were evaluated in lipopolysaccharides (LPS)-induced RAW264.7 macrophages model. After that, GC-MS analysis of the most active fraction was conducted for qualitative and quantitative estimation of its chemical composition, followed by a bioassay-guided fractionation to isolate its major active constituents, and explore their potential biological activities. Additionally, the potential binding modes and interactions of the isolated compounds with AChE and COX-2 active sites were studied using molecular docking to investigate better the mode of interaction underlying the inhibitory effects for the first time. Further, ADME computational parameters were studied *in silico* to predict the physicochemical properties of the isolated compounds. Finally, a molecular dynamic simulation was carried out to predict the performance of the isolated compounds upon binding to the active sites of AChE and COX-2, as well as their interaction and stability through the simulation. These findings may claim the multi-targeted potential of *A. pyrethrum* and its isolates against AD for the first time.

## Materials and methods

### Plant material

Akarkara (*Anacyclus pyrethrum* L.) roots were obtained from a local market (Harraz for Food Industry and Natural Products, Egypt) in March 2021 and authenticated by Mrs. Teresa Labib, Head of the Taxonomists at Orman Botanic Garden. This study complies with local, national, and international guidelines, and no specific consent was required for the collection of the plant material.

### Preparation and fractionation of the methanolic extract (ME)

The preparation and fractionation of the methanolic extract (ME) were done according to a previously published method with slight modifications [20]. Akarkara roots (5 kg) were dried, powdered, and extracted with methanol in soxhlet apparatus. The ME was evaporated under reduced pressure by vacuum distillation at a temperature not exceeding 50 °C. The residue (350 g) was suspended in water and subjected to liquid-liquid fractionation with methylene chloride (5 × 500 mL) followed by *n*-butanol saturated with water (5 × 500 mL). The collected solvent was evaporated under reduced pressure at a temperature not exceeding 50 °C to yield methylene chloride (MCF) (50 g) and butanol (BF) (44 g) fractions, respectively. The MCF was subjected to further bioassay-guided isolation procedures.

### Isolation of the major compounds from the methylene chloride fraction (MCF)

The MCF (30 g) was chromatographed on silica gel H 60 (500 g) using a vacuum liquid chromatography column (VLC) (25 L ҳ 7 D cm). The detailed chromatographic fractionation and isolation scheme is illustrated in Fig. S2. The details of the purification and isolation of each compound are recorded in the supplementary.

### Methods

The detailed procedures are described in the Supplementary online resource.

## Results and discussion

### Antioxidant and metal chelating activity of ME, MCF, and BF

**Fig. 1:**
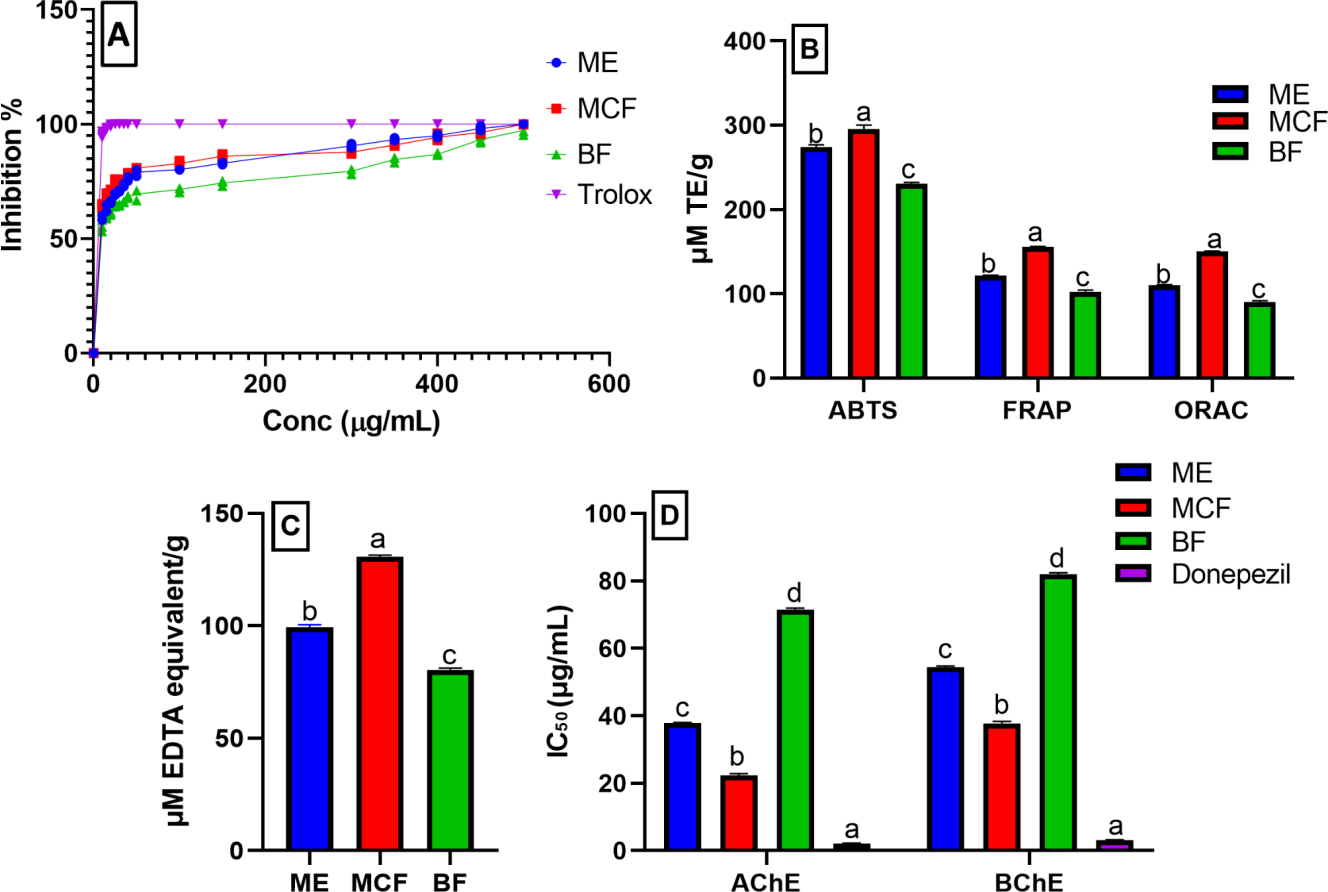
Antioxidant activity of *A. pyrethrum* roots methanolic extract (ME), methylene chloride (MCF), and butanol (BF) fractions using (**A**) DPPH represented by a line graph, (**B**) ABTS, FRAP, and ORAC, (**C**) metal chelating assay, and (**D**) anticholinergic activity (AChE and BChE). µM: micromolar; TE: Trolox; Data are expressed as mean ± standard deviation of three replicates; equivalent. Different letters on the bar indicate significant differences at *P* < 0.0001 with Tukey’s test

The generation of free radical species by oxidative stress as well as metal dyshomeostasis are crucial factors in the development of AD. Thus, antioxidants may help to prevent these processes, supporting anti-AD medication. The antioxidant, as well as metal chelating activity of the ME, MCF, and BF were assessed using five different in vitro assays viz.; radical scavenging activity [DPPH, ABTS, redox potential (FRAP), ORAC, and metal chelating assay (Fig. 1A-C). The IC_50_ values of the tested samples were determined using the DPPH assay using Trolox as a standard (IC_50_ 1.41 ± 0.08 µg/mL). Herein, the MCF showed the highest antioxidant potential with IC_50_ 10.60 ± 0.1 µg/mL, followed by the ME and BF (IC_50_ 13.78 ± 0.2 and 17.73 ± 0.16 µg/mL, respectively). The results were in accordance with previous literature determining the IC_50_ of *A. pyrethrum* methanolic extract (12.38 ± 0.25 µg/mL) [21] and was higher than that reported for 50% methanolic extract (IC_50_ 467.1 µg/mL) [22]. In addition, the MCF exhibited the highest antioxidant potential equivalent to 295 ± 5.14 µM TE/g (ABTS), 155.3 ± 0.70 µM TE/g (FRAP), 150.6 ± 0.32 µM TE/g (ORAC). Also, it showed the highest metal chelating activity explaining 130.7 ± 0.72 µM EDTA equivalent/g. These results suggested that MCF might have potential preventive values against AD and other oxidative-induced diseases. The in vivo antioxidant potential of the plant is well documented in literature [23]. Its antioxidant potential has been correlated with its traditional uses as anticonvulsant, brain tonic [24], anti-inflammatory [25], preventing oxidative DNA damage and cytotoxicity [22], anti-diabetic [26]. Further investigation could potentially lead to the discovery of phytochemicals with promising antioxidant and anti-neurodegenerative activities.

### Anticholinergic activity of ME, MCF, and BF

The main cholinesterase enzymes that significantly contribute to the hydrolysis and control of acetylcholine are AChE and BChE. Thus, inhibition of these enzymes increases the level of acetylcholine in the brain. In addition, numerous studies have highlighted the chaperone function of AChE through its peripheral anionic site in boosting the neurotoxicity of amyloid-*β* (Ab) fibrils and promoting their production [9]. Therefore, ChE inhibitors that simultaneously block the peripheral anionic and catalytic sites of AChE as well as the catalytic activity of BChE may have a double benefit, enhancing cholinergic transmission and possibly delaying the production of the extracellular plaques [27]. There are numerous ongoing researches for the discovery of natural anticholinergic drugs from plant sources. As most of the drugs currently available for the treatment of Alzheimer’s disease, such as rivastigmine and galanthamine are derived from natural products. It is worth mentioning that the AChE inhibitory activity of *A. pyrethrum* roots ethanolic extract was previously reported, recommending its use as anticholinergic [27]. Thus, the AChE and BChE inhibitory activities of the ME, MCF, and BF were determined (Fig. 1D). Herein, the MCF exhibited the highest inhibition of both AChE and BChE with the lowest IC_50_ (concentration required to inhibit 50% of the enzyme activity) corresponding to 22.4 ± 0.45 and 37.75 ± 0.65 µg/mL, compared to donepezil (IC_50_ 2.15 ± 0.13 and 3.15 ± 0.18, respectively). This potent inhibition suggested that MCF may be a candidate for the treatment of Alzheimer’s disease. Further chemical investigation of its component metabolites might guide the discovery of various cholinesterase inhibitors.

### In vitro anti-inflammatory activity of ME, MCF, and BF

**Fig. 2:**
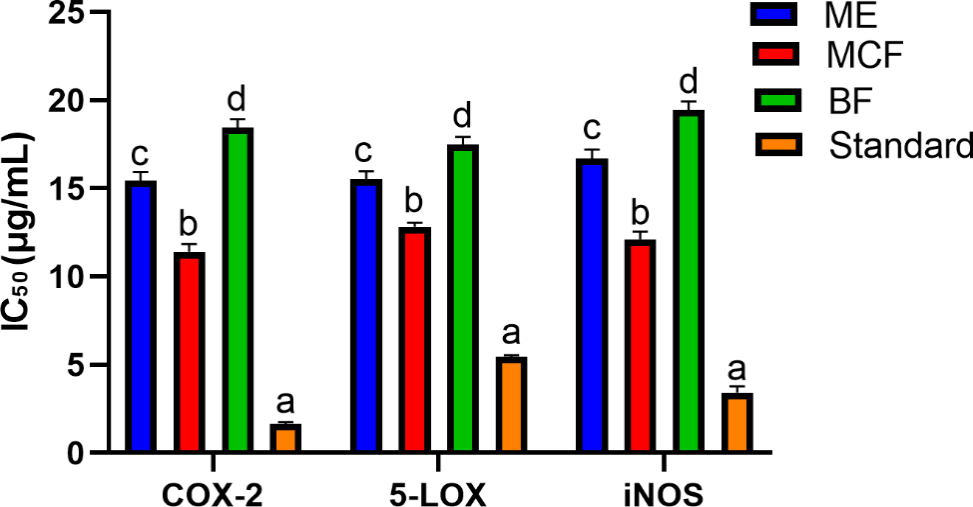
Anti-inflammatory activities of *A. pyrethrum* roots methanolic extract (ME), methylene chloride (MCF), and butanol (BF) fractions in vitro against COX-2 and 5-LOX, as well as in LPS-induced RAW264.7 macrophages (iNOS). Different letters on the bar indicate significant differences at *P* < 0.0001 with Tukey’s test. Standards: Celecoxib (COX 2), Zileuton (5-LOX), and parthenolide (iNOS) are serving as positive controls

Neuroinflammation is a major pathological aspect of AD as it leads to neurodegeneration. This is brought on by the increase of the inflammatory proteins (COX-2 and LOX), being expressed in the neuronal regions of the brains in AD patients [28]. Cyclooxygenase (COX-2) is a bi-functional enzyme that first catalyzes the addition of two molecules of oxygen to arachidonic acid to form the hydroperoxide, prostaglandin G_2_ (PGG_2_), and then reduces the latter to alcohol (PGH_2_), by peroxidase activity. Prostaglandins (PGs) are considered important biological mediators of inflammation, originating from the biotransformation of arachidonic acid catalyzed by cyclooxygenase. Thus, the inhibition of COX-2 enzyme will mediate anti-inflammatory actions by interfering with prostaglandin production [29, 30]. The concentration of tested samples causing 50% COX-2 inhibition (IC_50_) was calculated and compared to the standard Celecoxib (Fig. 2A). Moreover, the ability of the tested samples to inhibit 5-Lipoxygenase enzyme was investigated, which may have significant pro- or anti-inflammatory activity in AD. As, the lipoxygenases (LOXs) involve in the biosynthesis of leukotrienes and catalyze the addition of oxygen to linolenic, arachidonic (AA), docosahexaenoic acids (DHA), and other polyunsaturated fatty acids leading to the formation of bioactive lipids, significantly affect the course of neurodegenerative diseases [10]. However, inhibition of COX-2 shifts arachidonic acid metabolism from prostaglandins synthesis to leukotriene synthesis via upregulation of 5-LOX, resulting in increased levels of leukotrienes [31]. Therefore, dual COX-2/5-LOX inhibitors constitute an emerging therapy for inflammation. Thus, 5-LOX inhibitory activity of the tested samples was assessed in comparison to the standard Zileuton (Fig. 2A) and their IC_50_ values were determined.

The MCF showed the highest inhibition among tested samples against COX-2 (IC_50_ 11.4 ± 0.46 µg/mL) compared to Celecoxib (IC_50_ 1.65 **±** 0.10 µg/mL). It also exhibited the highest 5-LOX inhibitory potential (IC_50_ 12.82 ± 0.23 µg/mL) compared to the standard Zileuton (IC_50_ 5.44 ± 0.11 µg/mL). The results of the anti-inflammatory activity of MCF were correlated with its respective antioxidant and anticholinergic activities, which may aid in the development of novel therapies to relieve or prevent neurodegenerative disorders associated with AD.

### Cell viability on RAW264.7 macrophages

In MTT assay, the extract and fractions did not show cytotoxic activity (up to 250 µg/mL) (Figs. S4A and S5A-C), while the isolated compounds were safe up to 250 µM (Figs. S4B and S5D-G) when assayed on RAW264.7 macrophages (after 24 h incubation), indicating their safety and their potential as candidates for chronic treatment. The results suggest the ideal safety profile of *A. pyrethrum* extract and fractions, which match previous reports shedding light on their safety even for chronic treatments [32].

### LPS-induced RAW264.7 macrophages (iNOS) anti-inflammatory activity of ME, MCF, and BF

Macrophages are activated by different factors as pro-inflammatory cytokines and bacterial lipopolysaccharide (LPS). Activated macrophages produce many cytokines such as TNF-*α*, IL-6, and IL-1*β*, among other inflammatory mediators such as NO and PGE_2_ [10]. The free radical NO is produced by nitric oxide synthase (NOS), which exists as three isoforms: endothelial NOS (eNOS), inducible NOS (iNOS), and neuronal NOS (nNOS). LPS-stimulated macrophages produce NO by up-regulating iNOS expression through the production of inflammatory cytokines [10]. Thus, inhibition of these inflammatory mediators can be considered an effective strategy for the development of anti-inflammatory drugs. Regarding the inhibition of iNOS in the LPS-induced RAW264.7 macrophages (Fig. 2), MCF showed the highest inhibitory potential with IC_50_ equivalent to 12.11 ± 0.44 µg/mL compared to the standard drug; Parthenolide (IC_50_ 3.44 ± 0.35).

### Phytochemical characterization of the biologically active fraction (MCF)

#### Identification and quantification by GC-MS

As a result of the significant antioxidant, anticholinergic and anti-inflammatory activities of the MCF, compositional analysis of the MCF, was required to identify and quantify its compounds that could be correlated with its highest activities among the studied fractions. As the biological and pharmacological effects of non-polar fractions are frequently attributed to a variety of triterpenoid and steroid compounds [33]. However, these compounds are typically detected as complicated isomeric mixtures. It was proved that GC-MS is the method of choice for identifying triterpenes, sterols, glycerols, waxes, fatty acids, and other chemicals from non- and semi-polar fractions [34]. Several alkylamides have been reported in *Anacyclus* species. The structural resemblance of these amides makes it difficult to separate them from the mixtures. However, GC-MS is a powerful method for extracting known amides from mixtures [35]. Thus, GC-MS post silylation (Fig. S1) was employed for compositional analysis of the MCF. Forty-one compounds were identified and quantified (Table 1) representing 93.36% of the total compounds present. The detected compounds belong to different chemical classes, including terpenes, alkylamides, alkanes, fatty acids, esters, and sterols. Alkylamide was the major class detected, representing 59.59%, where oleamide constituted 17.53%, followed by 2E,4E-deca-2,4-dienoic acid 2-phenylethyl amide (11.22%), and deca-2E,4E-dienoic acid isobutylamide (pellitorine) (7.96%). Sterols represented the second major class, accounting for 12.33% with stigmasterol (8.78%) the predominant sterol. Previously, GC-MS characterization of the ethanolic extract of *A. pyrethrum* roots detected nearly similar classes of compounds including fatty acids, alkylamides, triterpenes, and sterols [36, 37]. Moreover, characterization of the hydroalcoholic extract of *A. pyrethrum* roots, seeds, leaves, and capitula showed the presence of various compounds as sarcosine, N-(trifluoroacetyl)-butyl ester, levulinic acid, malonic acid, palmitic acid, morphinan-6-one,4,5 *α*-epoxy-3-hydroxy-17-methyl, 2,4-undecadiene-8,10-diyne-N-tyramide, and isovaleric acid [38, 39]. The petroleum ether extract of *A. pyrethrum* roots was rich in fatty acids, esters and sterols [40]. To our knowledge, this is the first profiling and quantitative exploration of the methylene chloride fraction of *A. pyrethrum* roots rather than the total extract by GC-MS. The presence of diverse components in MCF of *A. pyrethrum* roots may be of great significance in various medicinal applications like in AD. Taking into consideration that, it is necessary to identify these compounds. Through previous studies, alkylamides were proved to exhibit antioxidant and anti-inflammatory activities, as well as being promising candidates for the treatment of neurological diseases such as Alzheimer’s. [41]. Previous reports revealed that terpenes (diterpenes, triterpenes and sesquiterpenes) are powerful cholinesterase inhibitors [42]. Triterpenes and sterols have been reported for their anti-inflammatory, anti-cholinesterase, and antioxidant activities, as well as their effectiveness against neurodegenerative disorders [43–45]. Furthermore, organic acids were proved to lower the incidence of neurodegenerative diseases and avoid the risk factors [46]. In addition, fatty acids showed effective management of AD through inhibition of neuroinflammation [47, 48]. As a result, these compounds may contribute to the previously determined antioxidant, anti-inflammatory, and anticholinergic activities of the most promising fraction (MCF).

**Table 1:** The relative percentage of silylated compounds in *A. pyrethrum* root methylene chloride fraction using GC-MS

RT (min)	RI	Compound	Molecular formula	Molecular weight	Relative %	SE	Chemical Class
13.41	1377	Copaene	C_15_H_24_	204	1.58	0.09	SC
13.75	1573	Spathulenol	C_15_H_24_O	220	0.40	0.12	SA
15.01	1613	Caryophyllene oxide	C_15_H_24_O	220	0.35	0.28	SO
15.21	1788	N-isobutyl-dodeca-2,4,8,10-tetraenamide	C_16_H_25_NO	247	0.76	0.99	AA
15.40	1802	Hexadecane	C_16_H_34_	226	0.49	0.89	A
15.91	1856	Heptadecane	C_17_H_36_	240	1.03	0.73	A
16.38	1878	N-isobutyl-2,4-heptadiene-6-monoynamide	C_11_H_15_NO	177	0.48	0.01	AA
16.76	1891	Lauric acid	C_12_H_24_O_2_	200	4.55	0.12	FA
16.96	1900	Myristic acid	C_14_H_28_O_2_	228	0.33	0.55	FA
17.09	1910	Pentadecanoic acid	C_15_H_30_O_2_	242	0.51	0.39	FA
17.61	1921	Palmitic acid	C_16_H_32_O_2_	256	1.56	1.01	FA
18.17	1936	Linoleic acid	C_18_H_32_O_2_	280	0.38	0.04	FA
18.45	1941	Stearic acid	C_18_H_36_O_2_	284	0.18	0.08	FA
19.02		Tetradeca-2E,4E-dien-8,10-diynoic acid isobutylamide (anacycline)	C_18_H_25_NO	271	0.30	0.12	AA
19.87	1946	Deca-2E,4E-dienoic acid isobutylamide (pellitorine)	C_14_H_25_NO	223	7.96	0.22	AA
20.20	1949	Deca-2E,4E,8Ztrienoic acid isobutylamide (8,9-(dehydropellitorine)	C_14_H_23_NO	221	2.33	0.67	AA
20.34	1951	Undeca-2E,4E-diene-8,10-diynoic acid isobutylamide	C_15_H_19_NO	229	0.49	0.78	AA
20.50	1959	Deca-2E,4E,8Ztrienoic acid piperidide	C_15_H_23_NO	233	0.86	0.89	AA
21.04	1976	Tetradeca-2E-diny-8,10-diynoic acid isobutylamide	C_18_H_27_NO	273	0.64	0.99	AA
21.84	1989	Tetradeca-2E,4E, nE-trienoic-8,10-diynoic acid Isobutylamide	C_18_H_23_NO	269	0.24	1.03	AA
23.02	2007	N-isobutyl-2,4-undecadiene-8,10-diynamide	C_16_H_21_NO	243	0.78	0.03	AA
23.11	2018	Dodeca-2E,4E, nE-trienoic acid 4-hydroxyphenylethylamide	C_20_H_27_NO_2_	313	0.46	0.06	AA
23.43	2029	2,8-N-isobutyl-2,8-dodecadienamide	C_16_H_29_NO	251	0.92	0.82	AA
23.59	2041	Tetradeca-2E,4E,8Etrienoic acid 4-hydroxyphenylethylamide	C_22_H_31_NO_2_	341	1.68	0.74	AA
25.99	2052	2E,4E-deca-2,4-dienoic acid 2-phenylethyl amide	C_18_H_25_NO	271	11.22	0.45	AA
26.12	2068	Deca-2E,4E-dienoic acid piperideide	C_15_H_23_NO	233	3.13	0.23	AA
26.30	2078	Deca-2E,4E,6Z-trienoic acid piperideide/Deca-2E,4E,6E-trienoic acid piperideide	C_15_H_21_NO	231	1.49	0.03	AA
26.40	2096	Deca-2E,4E,6Z,8Ztetraenoic acid piperideide/Deca-2E,4E,6E,8Ztetraenoic acid piperideide	C_15_H_19_NO	229	1.34	0.78	AA
26.52	2116	Tetradeca-2E,4E,12Ztriene-8,10-diynoic acid isobutylamide	C_18_H_23_NO	269	0.88	0.19	AA
26.67	2124	Phytol	C_20_H_40_O	296	0.44	0.34	D
26.87	2158	Linoleic acid ethyl ester	C_20_H_36_O	308	1.69	0.36	E
32.81	2200	Myristamide	C_14_H_29_NO	227.	1.51	0.21	AA
34.68	2275	Palmitoleamide	C_16_H_31_NO	253	1.13	0.58	AA
35.07	2377	Oleamide	C_18_H_35_NO	281	17.53	0.27	AA
35.23	2399	Stearamide	C_18_H_37_NO	283	3.46	0.13	AA
37.75	2410	Eicosane	C_20_H_42_	282	1.47	0.45	A
37.97	2734	Tetracosanoic acid, methyl ester	C_25_H_50_O	366	1.73	0.78	E
39.50	2837	Squalene	C_30_H_50_	410	2.12	0.60	T
39.77	3172	Stigmasterol	C_29_H_40_O	412	8.78	0.29	S
39.99	3353	γ- Sitosterol	C_29_H_50_O	414	3.55	0.78	S
40.46	3370	Lupeol	C_30_H_50_O	426	2.63	0.97	T
		**Terpenes (%)**					**7.52**
		**Alkylamides (%)**					**59.59**
		**Alkanes (%)**					**2.99**
		**Fatty acids (%)**					**7.51**
		**Esters (%)**					**3.42**
		**Sterols (%)**					**12.33**
		**Total identified components (%)**					**93.36**

Values are means ± standard deviations (𝑛=3). A: alkane; AA: alkylamide; D: diterpene; E: ester; FA: fatty acid; S: sterol; SA: sesquiterpene alcohol; SC: sesquiterpene hydrocarbon; SO: sesquiterpene oxide; T: triterpene; RI; retention index on DB-5-MS column relative to n-alkanes C8-C30.; R_t_: retention time; SE: standard error

#### Isolation of the major compounds

Extensive chromatographic fractionation and purification of the MCF (Fig. S2) was performed with the aim of isolating its major active compounds and evaluating their respective biological potentials, which is then demonstrated by detailed ADME studies. Four compounds (**A1-A4**) were isolated and identified by their physicochemical characters, NMR-spectroscopic analyses (^1^ H and ^13^ C), and comparison with the available literature (NMR details are in Supplementary material). The identities of the isolated compounds were confirmed as **A1**: 9-cis-octadecenamide (oleamide) [49], **A2**: stigmasterol [50], **A3**: 2E, 4E-deca-2,4-dienoic acid 2-phenylethyl amide [51] and **A4**: deca-2E,4E-dienoic acid isobutylamide (pellitorine) [52]. As far as the available literature is concerned, this is the first report on the isolation of oleamide from genus *Anacyclus*. The chemical structures of compounds **A1-A4** are illustrated in Fig. S3.

### Antioxidant and metal chelating activity of the isolated compounds (A1-A4)

**Fig. 3:**
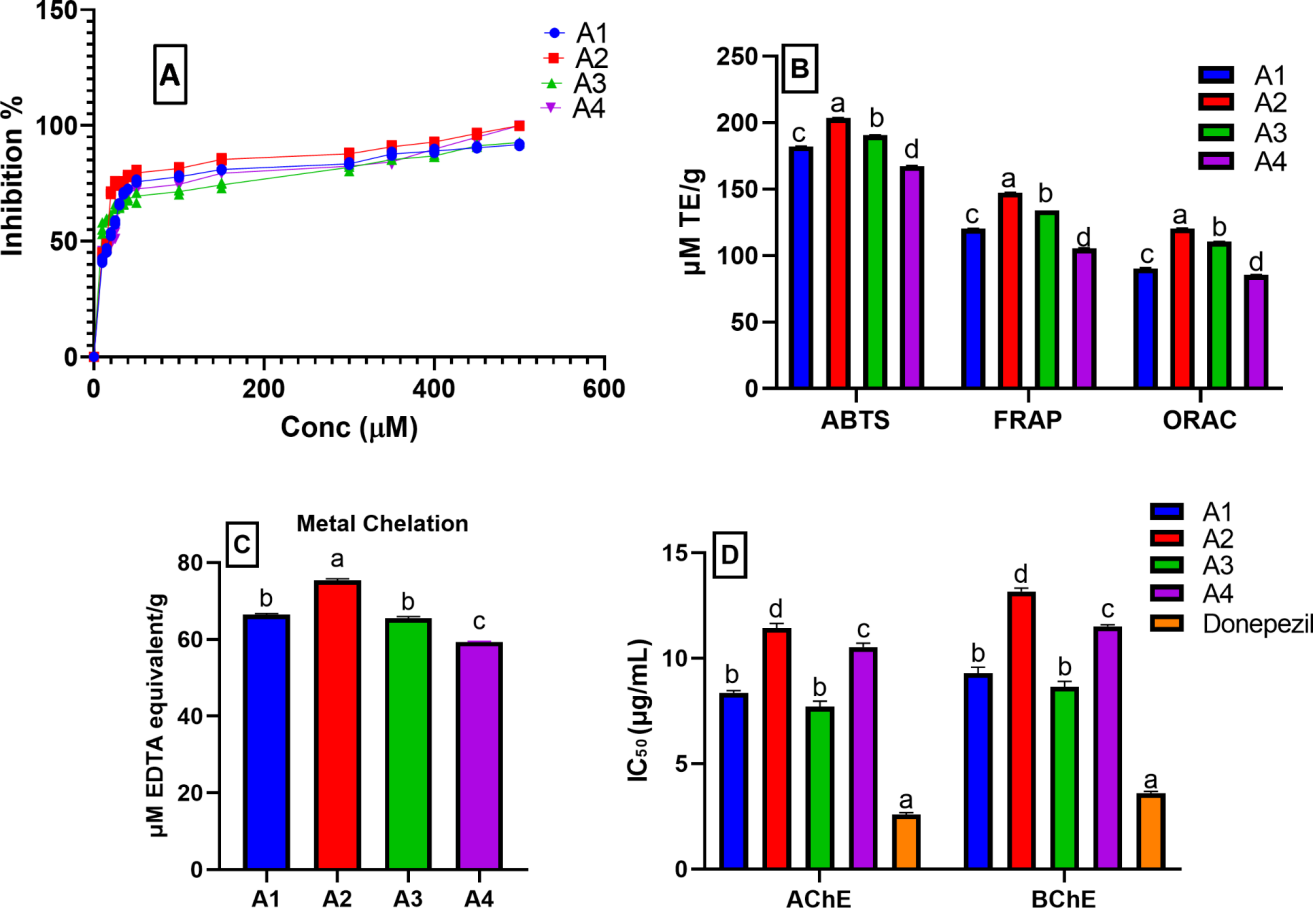
Antioxidant activity using (**A**) DPPH represented by a line graph, (**B**) ABTS, FRAP, and ORAC assays, (**C**) metal chelating assay, and (**D**) anticholinergic activity (AChE and BChE) of the isolated compounds (**A1-A4**). µM: micromolar; TE: Trolox equivalent. Data are expressed as mean ± standard deviation of three replicates; Different letters on the bar indicate significant differences at *P* < 0.05 with Tukey’s test

The DPPH radical scavenging assay determined the IC_50_ of the isolated compounds (Fig. 3A). Stigmasterol (**A2**) showed the highest activity with IC_50_ 16.37 ± 0.23 µM relative to Trolox (IC_50_ 5.63 µM), followed by 2E,4E-dienoic acid 2-phenylethylamide (**A3**) (IC_50_ 17.73 ± 0.16 µM), oleamide (**A1**) (IC_50_ 21.67 ± 0.21 µM), and pellitorine (**A4**) (IC_50_ 22.55 ± 0.41 µM). In addition, three extra assays; ABTS, FRAP, and ORAC were used to assess the isolated compounds’ antioxidant activity [53]. The four compounds exhibited high antioxidant potential ranging from 203.46 ± 0.31 to 85.51 ± 0.24 µM TE/g (Fig. 3B). Stigmasterol (**A2**) showed the highest antioxidant potential followed by deca-2E,4E-dienoic acid 2-phenylethylamide (**A3**), and oleamide (**A1**), then pellitorine (**A4**). The four isolated compounds showed high metal chelating activity (Fig. 3C) ranging from 75.36 ± 0.46 to 59.34 ± 0.19 µM EDTA equivalent/g. The results were in accordance with previous reports describing the antioxidant activity and metal chelating activity of alkylamides [54]. Moreover, the results are compatible with the previously reported antioxidant potentials of fatty amides and stigmasterol [55].

### Anticholinergic activities of the isolated compound (A1-A4)

The four isolated compounds showed powerful anticholinergic activities against AChE and BChE (Fig. 3D). Among the isolated compounds, **A3** and **A1** showed the highest AChE inhibitory potential with IC_50_ 7.70 ± 0.26 and 8.37 ± 0.10 µM, respectively compared to the standard Donepezil (IC_50_ 2.59 ± 0.09 µM). Moreover, they were the most potent BChE inhibitors (IC_50_ 8.65 ± 0.25 and 9.29 ± 0.29 µM, respectively) relative to Donepezil (IC_50_ 3.60 ± 0.08 µM). It has been reported that alkylamides have marked AChE inhibitory activities and that is in agreement with our results [56]. It is worth noting that, the inhibitory effects of **A1** and **A3** were comparable. Herein, the in vitro AChE and BChE inhibitory potential of oleamide (**A1**) was revealed for the first time.

### Anti-inflammatory activities of the isolated compounds (A1-A4)

**Fig. 4:**
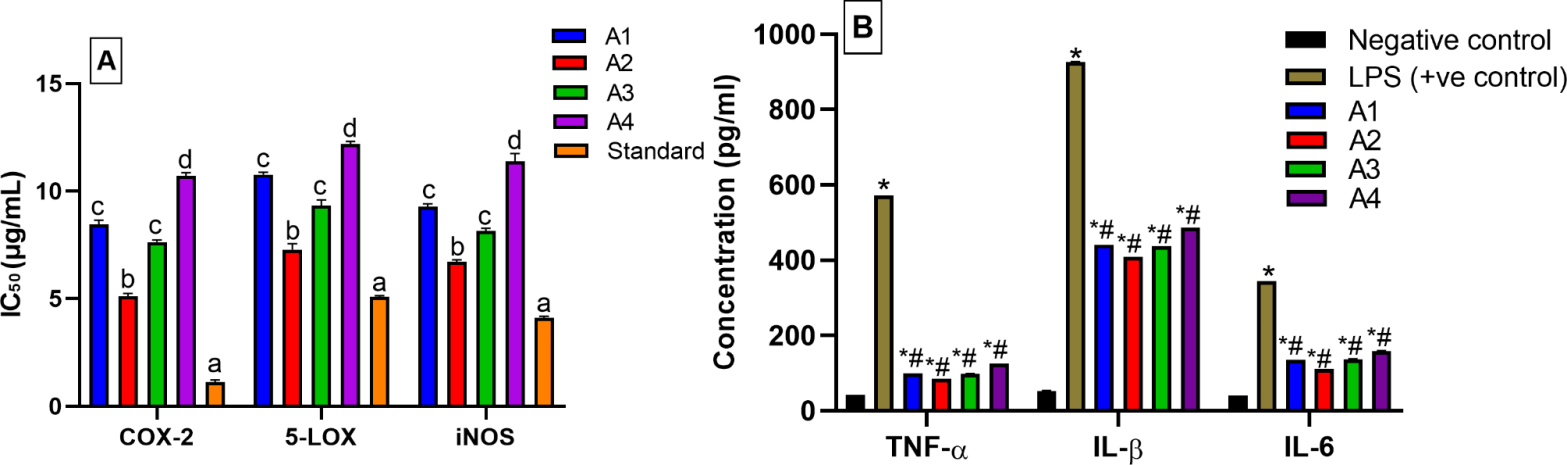
(**A**) In vitro COX-2 and 5-LOX, as well as in LPS-induced RAW264.7 macrophages (iNOs) anti-inflammatory activities, and (**B**) the inhibitory effect of pro-inflammatory cytokines (TNF- *α*, IL-1*β*, and IL-6) secretion in LPS-stimulated RAW264.7 macrophage cells of the isolated compounds (**A1-A4**). Different letters on the bar indicate significant differences at *P* < 0.0001. * Significant from negative control at *P* < 0.0001. ^#^ Significant from positive control at *P* < 0.0001 with Tukey’s test

The in vitro inhibitory effects of the isolated compounds on the inflammatory enzymes COX-2 and 5-LOX were evaluated (Fig. 4A). The isolated compounds showed remarkable COX-2 inhibitory activities ranging from 5.11 ± 0.12 to 10.70 ± 0.15 µM compared the standard Celecoxib (IC_50_ 1.13 ± 0.08 µM). The isolated compounds exhibited also powerful 5-LOX inhibitory potentials ranging from 7.280 ± 0.27 to 12.18 ± 0.12 µM relative to the standard Zileuton (IC_50_ 5.08 ± 0.06 µM). Interestingly, **A2** had the highest COX-2 and 5-LOX inhibitory activities, followed by **A3** and **A1**, and finally, **A4**, which had the least effect on the tested enzymes. These results are supported by previous studies highlighting the anti-inflammatory activities of alkylamides [38], stigmasterol [57], and oleamide [58]. This study explores the anti-inflammatory activities of the isolated compounds and ascertains that they can potentially combat inflammation which results in exacerbating AD.

### Effect of the isolated compounds (A1-A4) on iNOS activity in LPS-induced RAW264.7 macrophages

The isolated compounds inhibited iNOS in LPS-induced RAW264.7 macrophages (Fig. 4A), with IC_50_ values ranging from 6.72 ± 0.08–11.38 ± 0.36 µM compared to the standard drug Parthenolide (IC_50_ = 4.10 ± 0.08 µM). Stigmasterol (**A2**) was the most active (IC_50_ 6.72 ± 0.08 µM), followed by **A3** and **A1** with IC_50_ values of 9.15 ± 0.12 µM and 10.29 ± 0.12 µM, respectively. While **A4** appeared to be the least active and had IC_50_ of 11.38 ± 0.36 µM. So far, the four isolated compounds seemed to have anti-neuroinflammatory potential fighting the most important risk factor for AD and providing a new strategy for the development of anti-AD drugs.

### Effect of the isolated compounds (A1-A4) on pro-inflammatory cytokines production

The anti-inflammatory activity of the isolated compounds (**A1-A4**) was justified in a cell-based assay using RAW264.7 macrophages. The secretion of TNF-*α*, IL-1*β*, and IL-6 increased after treatment with LPS alone but the co-treatment with LPS and each isolated compound significantly decreased the pro-inflammatory cytokines production (*p* < 0.0001) compared to the positive control (Fig. 4B). Depending on the current results, the four compounds are appreciated as anti-inflammatory agents with resemble effects. These results were justified by published studies showing the potential effects of oleamide [59], stigmasterol [57], and pellitorine [29]. These results report, for the first time, the promising inhibitory activity of deca-2E,4E-dienoic acid 2-phenylethylamide (**A3**) against the pro-inflammatory cytokines in LPS-induced RAW264.7 macrophages. Accordingly, these results suggested that the anti-inflammatory effect of MCF could be associated with the suppression of NO production and iNOS expression through the down-regulation of TNF-*α*, IL-1*β*, IL-6, and COX-2. Best of our knowledge, this is the first study that evaluated the combinative effects of Akarkara root-derived metabolites with anti-inflammatory, antioxidant, and anticholinesterases effects. Our results highlighted the health benefits of such a traditional remedy that possesses various meritorious properties for drug discovery. It could treat neurodegenerative diseases by different mechanisms, including alleviation of oxidative stress, inhibition of inflammatory processes, reducing plaque formation, and preventing neural cell apoptosis.

### *In silico* studies

#### Molecular docking

**Fig. 5:**
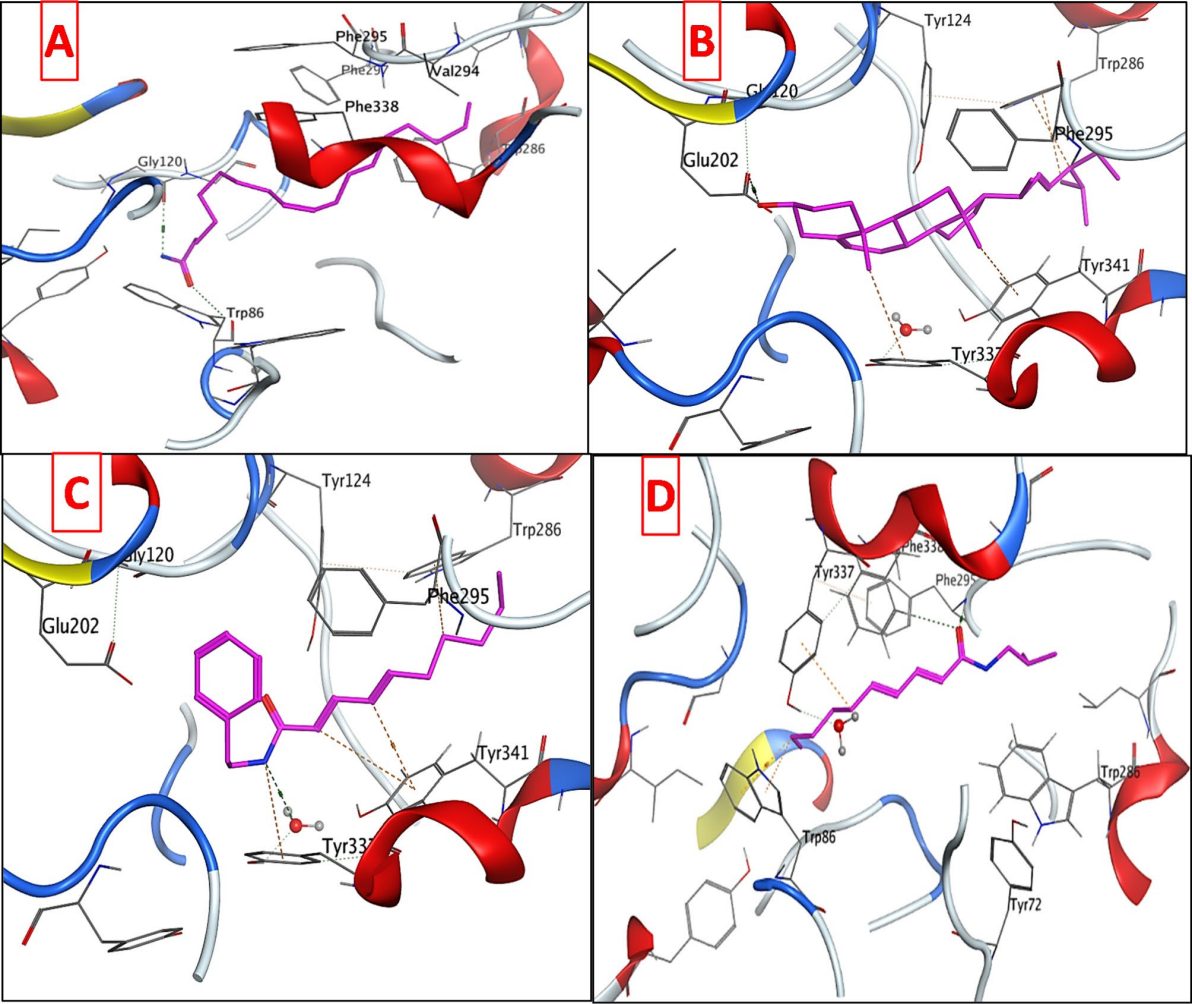
3D interaction diagrams of (**A**) oleamide (**A1**), (**B**) stigmasterol (**A2**), (**C**) deca-2E,4E-dienoic acid 2-phenylethylamide (**A3**), and (**D**) pellitorine (**A4**) in AChE binding site

To explore the potential anticholinergic activity of the isolated compounds; oleamide (**A1**) stigmasterol (**A2**), deca-2E,4E-dienoic acid 2-phenylethylamide (**A3**), and pellitorine (**A4**), they were tested for their inhibitory activity against AChE and BChE. The isolated compounds displayed pronounced activities against both enzymes. Consequently, a molecular docking study was performed for the mentioned compounds using AChE as a target protein to rationalize their promising inhibitory activity and to predict their plausible binding modes. **A1, A2, A3**, and **A4** were docked into the three-dimensional X-ray crystallographic structure of AChE (PDB code: 4EY7) [60] in complex with Donepezil. The three subsites that make up the AChE active site are a peripheral anionic site (PAS), which contains Phe295, Asp74, Tyr124, and Trp286 residues, a mid-aromatic gorge, and a catalytic active site (CAS) made up of the amino acids Gly448, Glu202, Tyr337, and Trp86. Clearly, the anti-Alzheimer drug Donepezil forms water mediated hydrogen bonds with Tyr337 and Asp74 via its positively charged NH and a hydrogen bond with Phe295 through its carbonyl moiety. Moreover, pi-stacking interactions are observed with Trp86 and Trp286 (Fig. S6). Docking setup was first validated by self-docking of the native ligand, Donepezil, into the active site of AChE. The validation results disclosed that the docking methodology is appropriate for the proposed docking study. This was supported by the fact that Donepezil coordinates and its self-docked pose were perfectly aligned displaying a root mean square deviation (RMSD) of 0.1402 Å and binding affinity (S) of -9.1833 Kcal mol^− 1^. Additionally, the self-docked pose was able to accomplish the same binding interactions with the essential amino acids in AChE binding site as the co-crystallized ligand (Fig. S7). Examining the top docking poses of the four tested compounds revealed that oleamide (**A1**) was able to achieve two hydrogen bonds with Gly120 and Trp86 in the CAS through its amide moiety. Stigmasterol (**A2**) displayed a strong hydrogen bond with Glu202 in the CAS via its hydroxyl group. Some hydrogen-π interactions were also observed with the aromatic moieties of Trp286 in the PAS and Tyr341. The NH of deca-2E,4E-dienoic acid 2-phenylethylamide (**A3**) was engaged in water-mediated hydrogen bond with Tyr337 in the CAS in a similar mode to the co-crystallized ligand Donepezil, in addition to several hydrogen-π interactions with the aromatic moieties of Trp286 in the PAS, Tyr337, and Tyr341. The carbonyl group of pellitorine (**A4**) formed a hydrogen bond with Phe295 in the PAS like Donepezil and another hydrogen bond with Phe338. Besides, the docked compound showed two hydrogen-π interactions with Trp86 and one hydrogen-π interactions with Tyr337 (Fig. 5), it is worth mentioning that both amino acid residues exist in the CAS. Results of the molecular docking study (Fig. 5) of compounds **A1-A4** and Donepezil in the vicinity of AChE were recorded in Table 2. Overall, the docked compounds fit well in the active site of AChE and were able to occupy the catalytic active site and peripheral anionic site of the enzyme, achieving comparable docking scores to that of the native ligand. Compounds **A1** and **A3** displayed the highest docking scores (binding energies) reaching about (79%) of that of Donepezil. These results are well correlated with the respective in vitro assessment of the anticholinergic activity of the isolated compounds.

**Table 2:** Molecular docking data of the tested compounds in the AChE active site

Compound	S (Kcal mol^− 1^)	Amino acids	Interacting groups	Type of interaction	Length
Oleamide (**A1**)	-7.2476	Trp86 Gly120	C = O NH_2_	H-bond acceptor H-bond donor	3.21 2.86
Stigmasterol (**A2**)	-6.4568	Tyr337 Tyr341 Trp286 Trp286 Glu202	CH_3_ CH_3_ CH_3_ CH OH	H-Pi interaction H-Pi interaction H-Pi interaction H-Pi interaction H-bond donor	3.87 3.51 3.9 3.44 2.84
Deca-2E,4E-dienoic acid 2-phenylethylamide (**A3**)	-7.2342	Tyr337 Tyr337 Tyr341 Tyr341 Trp286	NH NH =CH =CH CH_2_	H_2_O-mediated H-bond H-Pi interaction H-Pi interaction H-Pi interaction H-Pi interaction	2.93 4.28 4.54 3.72 4.76
Pellitorine (**A4**)	-6.6189	Tyr337 Phe295 Phe338 Trp86 Trp86	CH_2_ C = O C = O CH_2_ CH_2_	H-Pi interaction H-bond acceptor H-bond acceptor H-Pi interaction H-Pi interaction	4.03 3.22 3.82 3.86 4.29
Donepezil	-9.1833	Asp74 Tyr337 Tyr341 Phe295 Trp286 Trp86	NH^+^ NH^+^ CH_2_ C = O Phenyl Phenyl	H_2_O-mediated H-bond H_2_O-mediated H-bond H-Pi interaction H-bond acceptor Pi-Pi interaction Pi-Pi interaction	2.88 2.88 3.64 3.28 3.80 3.94

The isolated compounds were also examined for their potential anti-inflammatory activity by performing enzyme inhibitory assay against COX-2, 5-LOX, and iNOS. The most promising results were attained against COX-2. Thus, the four tested compounds were docked in the vicinity of COX-2 binding site to justify their potency. The three-dimensional X-ray crystallographic structure of COX-2 (PDB code: 3LN1) [61] in complex with the Celecoxib was retrieved from the protein data bank. It is worth mentioning that the amino group of Celecoxib can donate three hydrogen bonding interactions with Ser339, Leu338, and Gln178 (Fig. S8). Additionally, another hydrogen bond is observed between the sulfonyl group and Arg499. Moreover, the validation outcomes demonstrated that the co-crystallized ligand could dock properly in COX-2 active site with root mean square deviation (RMSD) of 0.0903 Å and docking score (S) of -9.6345 Kcal mol^− 1^ (Fig. S9).

**Fig. 6:**
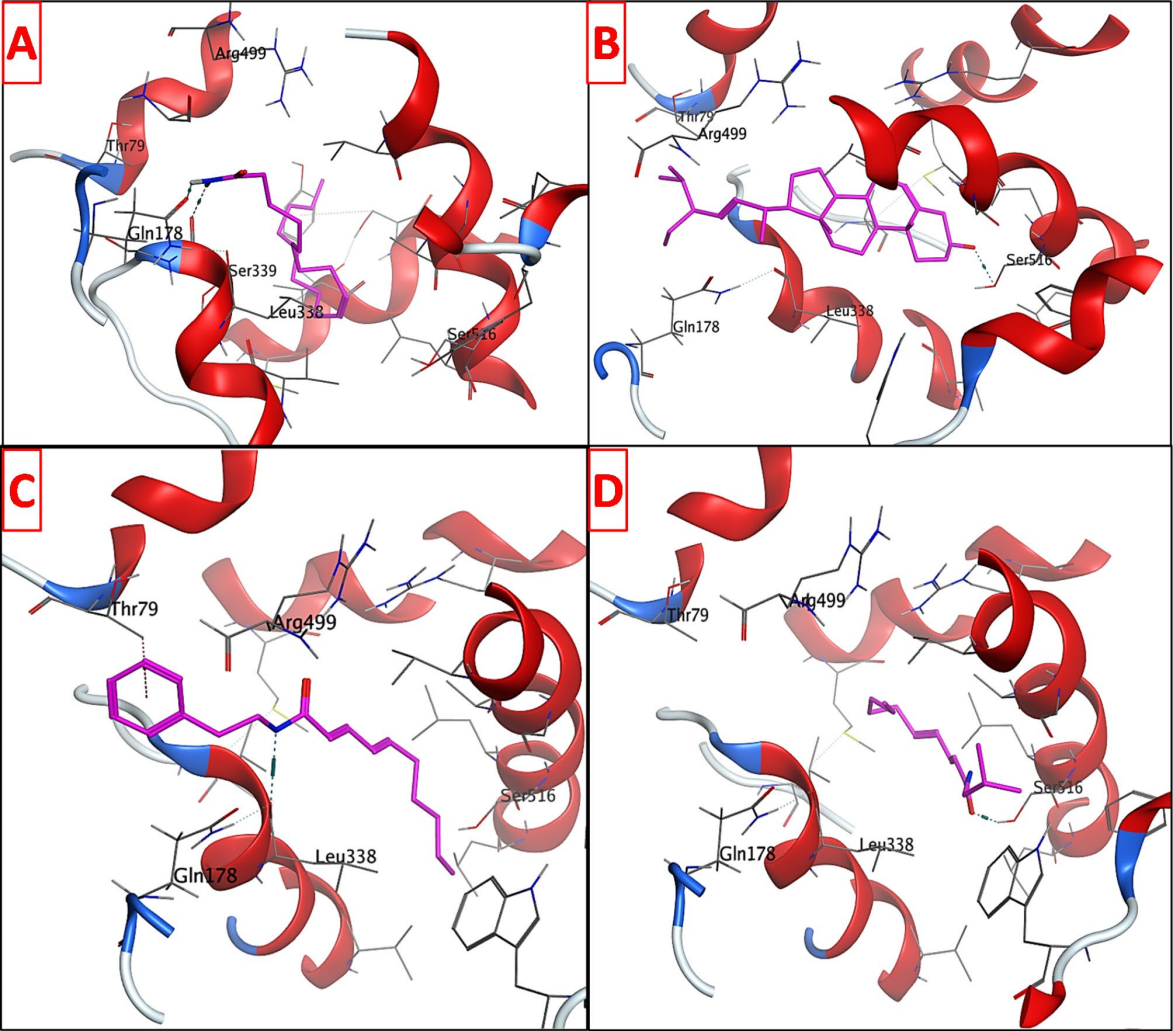
3D interaction diagrams of (**A**) oleamide (**A1**), (**B**) stigmasterol (**A2**), (**C**) deca-2E,4E-dienoic acid 2-phenylethylamide (**A3**), and (**D**) pellitorine (**A4**) in COX-2 binding site

Investigation of the docking data showed that the four docked compounds were able to achieve higher binding scores compared to the native ligand Celecoxib (Table 3). Oleamide (**A1**) donated two hydrogen bonding interactions to Ser339 and Gln178 similar to Celecoxib. Stigmasterol (**A2**) and pellitorine (**A4**) attained hydrogen bonds with Ser516 via their hydroxyl and carbonyl moieties, respectively, which could enhance the COX-2 inhibitory activity [62]. Moreover, deca-2E,4E-dienoic acid 2-phenylethylamide (**A3**) exhibited nearly similar orientation in the binding site of COX-2 through one hydrogen bonding interaction with leu338 and an arene-π interaction with Thr79. The results are represented in Fig. 6 and Table 3.

**Table 3:** Molecular docking data of the tested compounds in COX-2 active site

Compound	S (Kcal mol^− 1^)	Amino acids	Interacting groups	Type of interaction	Length
Oleamide (**A1**)	-11.7343	Ser339 Gln178	NH_2_ NH_2_	H-bond donor H-bond donor	2.96 3.03
Stigmasterol (**A2**)	-14.6288	Ser516	OH	H-bond donor	2.61
Deca-2E,4E-dienoic acid 2-phenylethylamide (**A3**)	-11.4957	Leu338 Thr79	NH Phenyl	H-bond donor H-Pi interaction	2.91 3.86
Pellitorine (**A4**)	-10.8175	Ser516	C = O	H-bond acceptor	2.68
Celecoxib	-9.6345	Ser339 Leu338 Gln178 Arg499	NH_2_ NH_2_ NH_2_ SO_2_	H-bond donor H-bond donor H-bond donor H-bond acceptor	2.93 3.04 3.06 3.54

The anti-inflammatory capacity of the Akarkara root against cognitive deficits and neurodegenerative conditions that are characteristics of AD, was assumed to be associated mainly with its nonpolar composition (Fig. 2). Thus, the interactions of the compounds isolated from the MCF with COX-2 receptor were investigated *in silico via* molecular docking. The four compounds docked within the COX-2 receptor gave binding energy values ranging from 10.8175 Kcal mol^-1^**(A2)** to 14.6288 kcal mol-1 **(A4)**. Thus, the remarkable binding affinities of the isolated compounds and their promising binding modes and interactions in the vicinity of COX-2 could justify the significant in vitro inhibitory activity of the investigated parent extract and its fractions in comparison to Celecoxib, supporting the possibility of a synergistic anti-inflammatory effect exerted by the isolated compounds. Finally, the current findings support the notion that some plants have better prospects as neurodegenerative protectors due to their potential anticholinergic and anti-inflammatory properties.

### *In silico* ADME profile and BBB permeability prediction

**Fig. 7:**
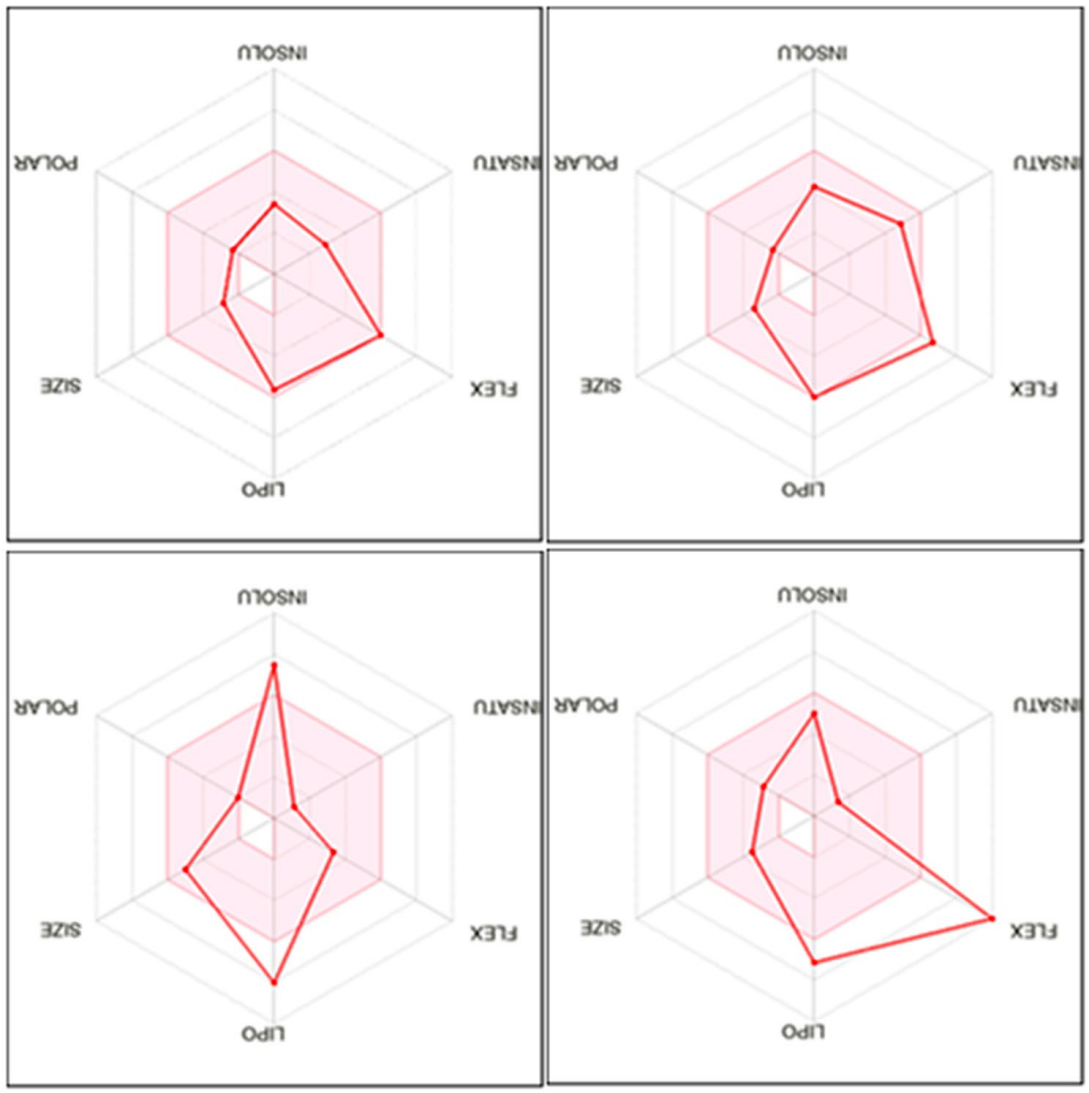
Bioavailability radar chart of the isolated compounds; oleamide (**A**), stigmasterol (**A2**), deca-2E,4E-dienoic acid 2-phenylethylamide (**A3**), and pellitorine (**A4**), respectively. The pink zone signifies the range of the optimal property values for oral bioavailability and the red line is the compounds’ predicted properties. Saturation (INSATU), size (SIZE), polarity (POLAR), solubility (INSOLU), lipophilicity (LIPO), and flexibility (FLEX)

The pharmacokinetics, physicochemical, and drug likeness properties of the four isolated compounds were predicted using the free accessible web server Swiss ADME (http://www.swissadme.ch/index.php). The outcomes of Swiss ADME prediction (Table 4) demonstrated that the examined compounds accomplished a predicted log Po/w value in the range of 3.64–6.98 and revealed no alerts for Pan Assay Interfering Substances (PAINS). Besides, three out of the four compounds were moderately water soluble, BBB permeable, and possessed high GIT absorption (for the boiled egg chart of the tested compounds see supporting materials, Fig. S10). All the tested compounds were not P-glycoprotein substrates. The Lipinski rule of five [63] and other models were used to determine the bioavailability scores. The molecular interactions involving cytochromes P450 isomers (CYP) were anticipated. Moreover, the oral bioavailability radar charts of the examined compounds displayed that they had good anticipated oral bioavailability as well as favorable pharmacokinetic features as depicted in Fig. 7.

**Table 4:** ADME properties of the isolated compounds predicted using SwissADME web server

Property	Oleamide (A1)	Stigmasterol (A2)	Deca-2E,4E-dienoic acid 2-phenylethylamide (A3)	Pellitorine (A4)
MW	281.48	412.69	271.4	223.35
Consensus LogPo/w	5.29	6.98	4.29	3.64
Log *S* (ESOL)	Moderately soluble	Poorly soluble	Moderately soluble	Soluble
#Rotatable bonds	15	5	10	9
#H-bond acceptors	1	1	1	1
#H-bond donors	1	1	1	1
MR	91.07	132.75	86.34	71.47
TPSA	43.09	20.23	29.10	29.10
Lipinski violations	1	1	0	0
Ghose violations	0	3	0	0
Veber violations	1	0	0	0
Egan violations	0	1	0	0
Muegge violations	1	2	1	0
Bioavailability score	0.55	0.55	0.55	0.55
PAINS alerts	0	0	0	0
Brenk alerts	1	1	2	2
Leadlikeness violations	2	2	2	3
GI absorption	High	Low	High	High
BBB permeant	Yes	No	Yes	Yes
P-gp substrate	No	No	No	No
CYP1A2 inhibitor	Yes	No	Yes	Yes
CYP2C19 inhibitor	No	No	Yes	No
CYP2C9 inhibitor	Yes	Yes	Yes	No
CYP2D6 inhibitor	No	No	No	No
CYP3A4 inhibitor	No	No	No	No

BBB: Blood–brain barrier; GI: Gastrointestinal; MR: Molar refractivity; P-gp: P-glycoprotein; PAINS: Pan Assay Interfering Substances; TPSA: Topological polar surface area

Crossing the blood-brain barrier is a necessity for drugs targeting the CNS. The inability of therapeutic molecules to penetrate the BBB is a key obstacle for CNS drug candidates and needs to be addressed quickly in the drug development process. Predicting the BBB permeability of new CNS drugs is therefore crucial. Accordingly, the BBB distribution for our candidates was additionally assessed using the web server pkCSM (http://biosig.unimelb.edu.au/pkcsm/prediction) in comparison with Donepezil as a reference drug [64]. The BBB permeability (blood-brain barrier permeability log BB) and CNS permeability (calculated using the blood-brain permeability surface area product log PS) are two *in silico* parameters provided by the pkCSM server. The first foretells a compound’s capability to cross the BBB. The second parameter does not account for the effects of the systemic distribution and is associated with the direct assessment of brain-blood permeability. Regarding the BBB permeability, all the tested compounds are readily able to cross the BBB, with values ranging from 0.811 to -0.397 in comparison with Donepezil (0.157). Considering the CNS permeability, the assessed compounds have high ability to penetrate the CNS with values ranging from 1.596 to -1.651 in comparison with Donepezil (-1.464). The calculated results are illustrated in Table 5.

**Table 5:** The central nervous system (CNS) distribution values of the isolated compounds calculated using pkCSM web server

Compound	Distribution	
	BBB permeability (log BB)^a^	CNS permeability (log PS)^b^
Oleamide (**A1**)	-0.397	-1.651
Stigmasterol (**A2**)	0.811	-1.033
Deca-2E,4E-dienoic acid 2-phenylethylamide (**A3**)	0.603	-0.976
Pellitorine (**A4**)	0.633	1.596
Donepezil	0.157	-1.464

^a^**log BB** should be > 0.3 and not less than − 1 for a compound to readily across the BBB

^b^**log PS** should be > -2 and not less than − 3 for a compound to penetrate the CNS

### Molecular dynamic and system stability

**Fig. 8:**
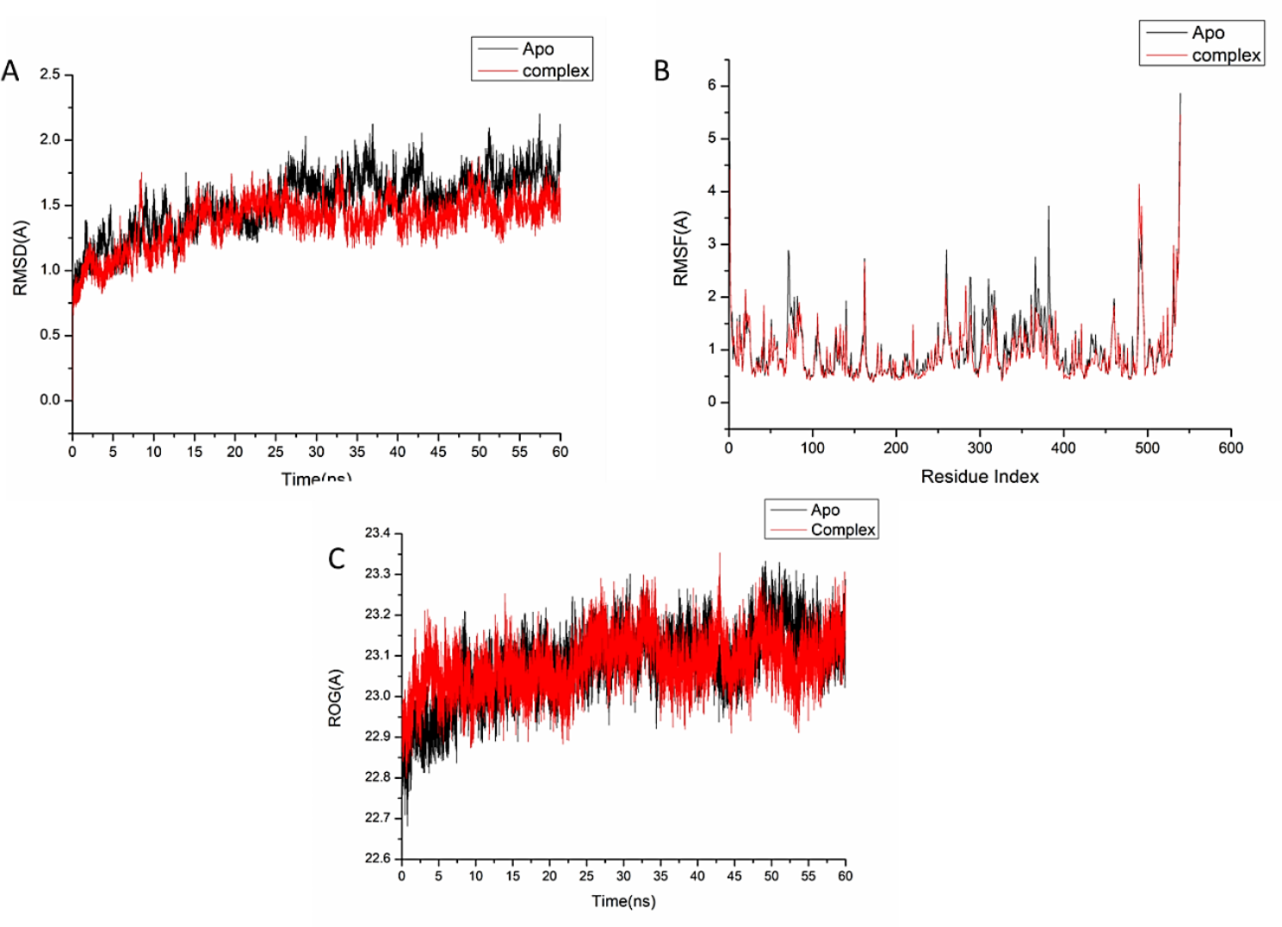
(**A**) RMSD of Cα atoms of the protein backbone atoms. (**B**) RMSF of each residue of the protein backbone Cα atoms. (**C**) RoG of Cα atoms of protein residues of the backbone atoms relative (black) to the starting minimized over 60 ns for the acetylcholinesterase receptor (AChE) receptor enzymes protein with oleamide (red)

**Fig. 9:**
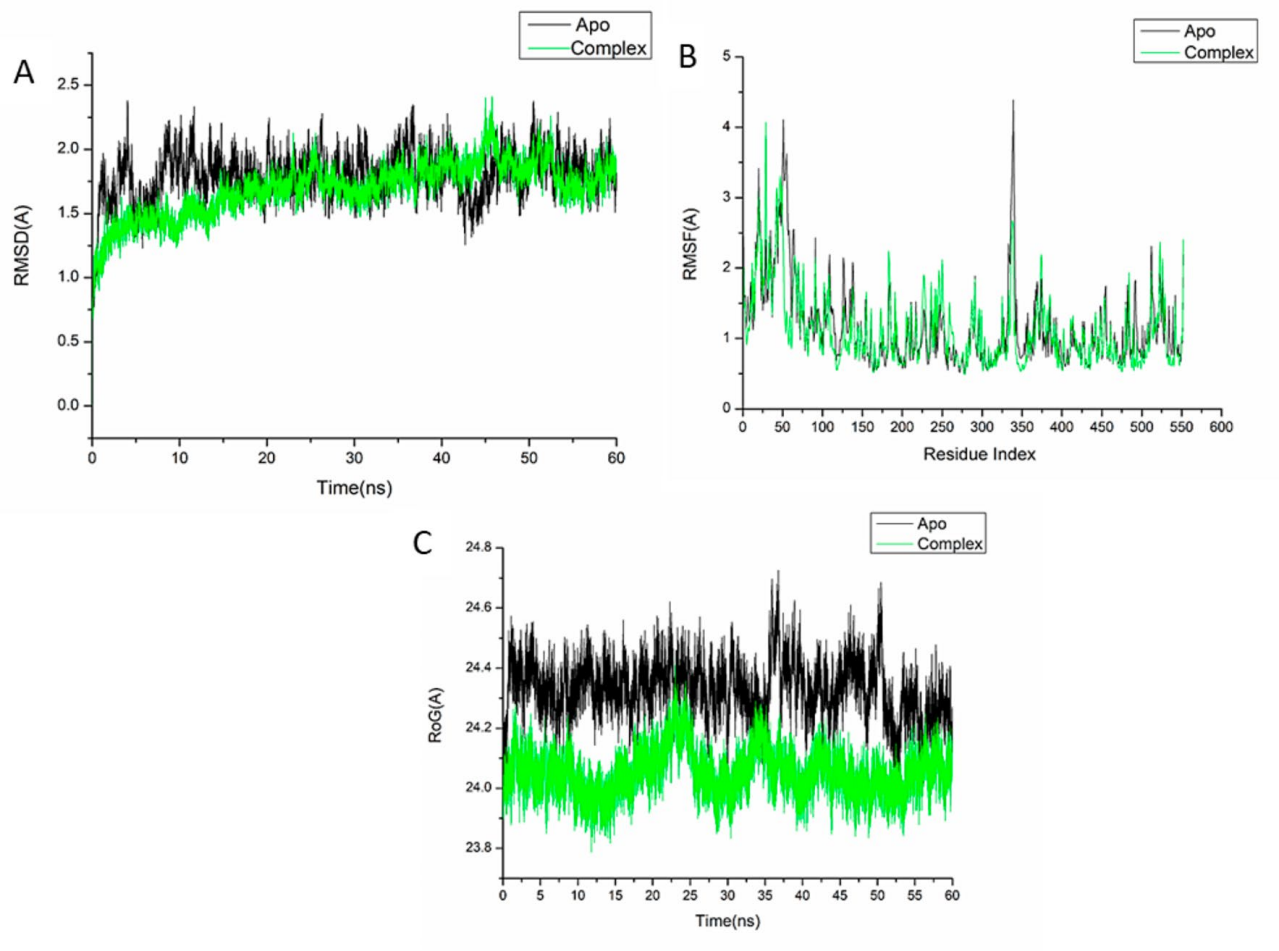
(**A**) RMSD of Cα atoms of the protein backbone atoms. (**B**) RMSF of each residue of the protein backbone Cα atoms (**C**) RoG of Cα atoms of protein residues of the backbone atoms relative (black) to the starting minimized over 60 ns for the cyclooxygenase-2 receptor enzymes protein with stigmasterol (green)

A molecular dynamic simulation was carried out to predict the performance of the isolated compounds upon binding to the active site of protein as well as their interaction and stability through the simulation [65, 66]. The validation of system stability is essential to trace disrupted motions and avoid artifacts that may develop during the simulation. This study assessed Root-Mean-Square Deviation (RMSD) to measure the systems’ stability during the 60 ns simulations. The recorded average RMSD values for the entire frames of the systems were 1.52 ± 0.22Å, and 1.37 ± 0.18 Å, for apo-AChE protein, and oleamide-AChE complex systems (Fig. 8A), and 1.80 ± 0.18Å,1.69 ± 0.21Å, for COX-Apo, COX-stigmasterol complex, respectively (Fig. 9A).

During MD simulation, assessing protein structural flexibility upon ligand binding is critical for examining residue behavior and their connection with the ligand [67]. Protein residues fluctuations were evaluated using Root-Mean-Square Fluctuation (RMSF) algorithm to evaluate the effect of inhibitor binding towards the respective targets over 60 ns simulations. The recorded average RMSF values for the entire frames of the systems were 1.02 ± 0.60Å, and 0.94 ± 0.54 Å, for apo-AChE protein, and oleamide-AChE complex systems (Fig. 8B), and 1.16 ± 0.60Å, 1.11 ± 0.53Å, for COX-Apo, COX-stigmasterol complex, respectively (Fig. 9B). These values revealed that the oleamide-, and stigmasterol-bound to protein complex systems had a lower residue fluctuation than the other systems.

The radius of gyration (Rg) is an indicator of protein structure compactness and stability during the simulation [68]. The recorded average RMSF values for the entire frames of the systems were 23.08 ± 0.08Å, and 23.07 ± 0.06 Å, for apo-AChE protein, and oleamide-AChE complex (Fig. 8C), and 24.33 ± 0.092Å, 24.05 ± 0.07Å, for COX-Apo, COX-stigmasterol complex, respectively (Fig. 9C). It was found that Rg of ligand-bonded protein exhibited a lower rigid structure than Apo-protein.

## Conclusion

This research shed light on *A. pyrethrum* as a multifunctional anti-Alzheimer drug in a first report. We can use the chemical profile of the methylene chloride fraction of *A. pyrethrum* L. roots obtained by GC-MS as a marker for its identification through elucidation of the main active constituents and establishing their proportions and the characteristic ratios between them. The isolated compounds showed potential antioxidant, anticholinergic, and anti-inflammatory activities. In this respect, the molecular docking and dynamic simulations with the key cholinergic (AChE) and inflammatory (COX-2) enzymes recommended these compounds as valuable multifactorial anti-Alzheimer drugs. Thus, further in vivo and clinical studies on *A. pyrethrum* and its isolated compounds are warranted to clarify their therapeutic potential as drug candidates targeting AD for clinical use.

### Electronic supplementary material

Below is the link to the electronic supplementary material.

Supplementary Material 1

## Data Availability

All data generated or analyzed during this study are included in this published article and its supplementary information file.

## Abbreviations

ABTS: 2,2'-azino-di(3-ethylbenzthiazoline-6-sulfonic acid
AChE: Acetylcholinesterase
AD: Alzheimer’s disease
BChE: Butyrylcholinesterase
COX: Cyclooxygenase
DPPH: 2,2-diphenyl-1-picrylhydrazyl
DW: Dried weight
FRAP: Ferric reducing antioxidant power
IC_50_: Half maximal inhibitory concentration
IL: Interleukin
LOX: Lipoxygenase
iNOS: Inducible nitric oxide synthase
BF: Butanol fraction
MCF: Methylene chloride fraction
ME: Methanolic extract
ORAC: Oxygen radical absorbance capacity
TE,: Trolox equivalent
TNF-α: Tumor necrosis factor alpha
