# Supplementary material for: Combinative effects of akarkara root-derived metabolites on anti-inflammatory and anti-alzheimer key enzymes: integrating bioassay-guided fractionation, GC-MS analysis, and in silico studies
Source: BMC Complement Med Ther. 2023 Nov 17;23:413. doi: 10.1186/s12906-023-04210-6 (PMC10655324; doi:10.1186/s12906-023-04210-6)
Supplement: Supplementary file 1 — Supplementary Material 1 [file 12906_2023_4210_MOESM1_ESM.docx]

**Supplementary Material**

**Combinative Effects of** **Akarkara Root-Derived Metabolites on Anti-inflammatory and Anti-Alzheimer Key Enzymes: Integrating Bioassay-Guided Fractionation, GC-MS Analysis, and *In Silico* Studies**

**Rana M. Ibrahim ^1,^**^†^**, Passent M. Abdel-Baki^1, *,^** ^†^**, Ghada F. Elmasry^2, *^,** **Ahmed A. El-Rashedy^3^, Nariman E. Mahdy^1^**

**^1^**Pharmacognosy Department, Faculty of Pharmacy, Cairo University, Kasr-El-Ainy Street, 11562, Cairo, Egypt

^2^Department of Pharmaceutical Chemistry, Faculty of Pharmacy, Cairo University, Kasr El-Aini Street, 11562 Cairo, Egypt.

**^3^**Natural and Microbial Products Department, National Research Center (NRC), Dokki 12622, Giza, Egypt.

*****Corresponding authors: Passent M. Abdel-Baki, [passent.mohamed@pharma.cu.edu.eg](mailto:passent.mohamed@pharma.cu.edu.eg)

Ghada F. Elmasry, [ghada.elmasry@pharma.cu.edu.eg](mailto:ghada.elmasry@pharma.cu.edu.eg)

† These authors contributed equally to this work.

**Material and Methods**

**General procedures**

For chromatographic isolation, silica gel 60 (70–230 mesh), silica gel RP-18, and precoated silica gel 60 F_254_ plates were purchased from Sigma-Aldrich Chemicals, Germany. For TLC development, solvent systems; S_1_: *n*-hexane-ethyl acetate (80:20 v/v) and S_2_: methylene chloride-methanol (97:3 v/v) were used. For visualization of chromatograms, spraying with *p*-anisaldehyde–sulphuric acid, followed by heating at 110°C. All the used solvents for extraction and fractionation were of analytical grade. For the determination of melting points (uncorrected), an electrothermal 9100 (UK) was utilized. For cyclooxygenase (COX) inhibitory activity, human recombinant COX-2 enzyme was used (Cayman Chemicals, USA). 5-LOX inhibitory screening assay kit was purchased from MyBioSource, USA. RAW264.7 macrophage cells were obtained from Shanghai BOGO Industrial Co., Ltd. (Shanghai, China), Dulbecco’s modified Eagle’s medium (DMEM), and fetal bovine serum (FBS) were supplied by Hyclone, General Electric Healthcare Life Sciences, (Mississauga, Canada). Penicillin streptomycin (P/S) was obtained from Solarbio life sciences, (Beijing, P. R. China). Lipopolysaccharide (LPS) and 3-(4,5-dimethylthiazol-2-yl)-2,5-diphenyl-2H-tetrazolium bromide (MTT) were purchased from Sigma-Aldrich Chemicals, Germany. IL-*β* (ab46052), IL-6 (ab178013), and TNF-*α* (ab181421) kits were purchased from (abcam®, Human ELISA kit; China). AChE (Electricell AChE, Type-VI-S, EC 3.1.1.7, Sigma) and butyrylcholinesterase BChE (horse serum BChE, EC 3.1.1.8, Sigma) were used for the cholinesterase inhibitory activity. The absorbances were measured using Tecan, microplate reader, ((Infinite F50, Switzerland)). The NMR spectra [^1^H-NMR (400 MHz) and ^13^C-NMR (100 MHz)] were determined by Bruker NMR system in CD_3_OH and DMSO-d6. Chemical shifts are represented in δ (ppm) relative to the internal standard TMS. The reagents used for biological investigations were purchased from Sigma Chemical Company (CA, USA).

**Antioxidant and metal chelating assays**

The extract and fractions were prepared in dimethyl sulfoxide (DMSO) at a concentration of 1mg/mL, while the isolated compounds were tested in 500 µM.

***DPPH assay***

The 2,2-diphenyl-1-picrylhydrazyl (DPPH) assay was performed as described previously [1]. One hundred μL of DPPH reagent (freshly prepared as 0.1% in methanol) was mixed with equal volume of each sample. Then, the solutions were left in the dark at room temperature for 1hour minutes. Trolox (6-hydroxy-2,5,7,8-tetramethylchroman-2-carboxylic acid) was used as a positive control. The absorbances were measured at 517 nm. Data are represented as means ± SD according to the following equation:

Percentage inhibition = [(Average absorbance of blank - average absorbance of the test)/(Average absorbance of blank)] x 100

The IC_50_ of each tested sample was determined.

***ABTS assay***

The capability of the tested samples to scavenge ABTS (2,2`-Azino-bis(3-ethylbenzothiazoline-6-sulfonic acid)) free radicals was carried out according to the previously published procedure [2]. ABTS radical cation (ABTS•+) was formed by the reaction of ABTS aqueous solution and potassium persulfate. The decrease of the formed ABTS color intensity was measured spectrophotometrically at 750 nm. A serial dilution of Trolox (positive control) was prepared (50–600 μM) to establish dose-response curves. The blank was made up of 200 μL of methanol. Results were expressed as micromolar (μM) Trolox equivalent (TE) per gram of tested sample (i.e., μM TE/g) using the linear regression equation extracted from the calibration curve of Trolox (linear dose-inhibition curve).

***FRAP assay***

The Ferric reducing antioxidant power (FRAP) assay depends on the reduction of Fe3+ to Fe2+, which is chelated by 2,4,6-tris(2- pyridyl)-s-triazine (TPTZ) to form Fe2+-TPTZ complex resulting in the formation of a blue-colored ferrous-probe complex from a colorless ferric-probe complex. The assays were performed accordingthe to previous method [3]. The absorbance of the blue color produced is measured at 593 nm at the end of the incubation time. Trolox was useda as positive control to establish a dose response curve (50-800 μM). A blank was done with the tested samples and control to eliminate the contribution of the color to the absorbances. Using the linear regression equation extracted from the standard calibration curve, the ferric reduction capacity of the samples is expressed as μM TE/ g sample (linear dose–response curve of Trolox).

***ORAC assay***

The Oxygen radical antioxidant capacity (ORAC) assay depends on the inhibition of peroxyl-radical-induced oxidation initiated by thermal decomposition of 2,2′-azobis (2-amidino-propane) dihydrochloride (AAPH). The assay was performed according to previously published procedures [3]. The fluorescence was recorded for 60 min at excitation and emission wavelengths of 485 and 530 nm, respectively. A blank was formed of 300 µL of phosphate buffer in the reaction mixture. Trolox was used as a positive control to establish a dose response curve (12.5-400 μM).

***Metal chelating assay***

The ability of the tested samples to chelate Fe^2+^ was determined according to the method described before [4]. The absorbances were recorded at 593 nm. Blank was formed by adding 50 μL of distilled water. A serial dilution of EDTA (positive control) was prepared in water to generate dose-response curves (5-80 *µ*M). Results are described as μM EDTA equivalent/gram of tested sample (μM EDTA eq/g) using the linear regression equation extracted from the calibration curve of the standard (linear dose-inhibition curve of EDTA).

**Anticholinergic activity**

***Acetyl- (AChE) and butyrlcholinesterase (BChE) Inhibition Assays***

The cholinesterase inhibitory activity was determined colorimetrically as previously published method [5] with slight modifications. Serial dilutions (in DMSO) of the tested extract, and fractions were (125- 0.98 µg/mL), while those of isolated compounds and standards were (250 - 0.98 µM). In a 96-well plate, 170 μl Tris-HCl buffer (200 mM, pH 7.5) was mixed with 20 μL of each tested sample solution and 20 μL of 0.1 U/ml enzyme solution (Ache or BChE) containing 0.1% bovine serum albumin in the same buffer. Dithio-bis-(2-nitrobenzoic acid) DTNB, an indicator, was added after 10 min of incubation at 25 °C (0.69 mM in tris buffer). The reactions were initiated by the addition of 20 μL (1.11 mM) of acetyl thiocholine iodide (ATCI) or butyrylthiocholine chloride (BTCl) for AChE and BChE assays, respectively. Donepezil was used as a standard drug. The intensity of the developed color was measured after 10 min at 405 nm (reading A). In the control assays without the inhibitors, the tested samples were replaced by DMSO (20 μl) and their absorbance values were recorded (Reading B). Regarding the blank assays, the buffer (20 μL) was added instead of the enzyme.

The % inhibition was calculated using the following formula:

% of inhibition = [1− (corrected A/corrected B)] * 100

**Anti-inflammatory activity**

***In vitro cyclooxygenase (COX-2) inhibitory activity***

Stock solutions of the tested extract and fractions (1 mg/mL) and isolated compounds (1000 µM) were prepared in (DMSO). Subsequent serial dilutions of the extract and fractions (125-0.98 µg/mL), as well as isolated compounds and standards (250-0.98 µM), were done in a 96-well plate. The inhibition COX-2 enzyme was assessed colorimetrically [6]. Blank was formed by the reaction mixture containing assay buffer and heme. The 100% initial activity (IA) was established by a mixture of COX-2 assay buffer and heme. Celecoxib served as positive control. The absorbances of oxidized TMPD (blue color) were determined in ELISA plate reader at 590 nm. The % inhibition was calculated using the following formula:

COX % Inhibition= [(100%IA−Inhibitor)/ 100% IA x 100]. Where IA: 100% initial activity.

The concentration at which the tested sample produces 50% inhibition of COX-2 (IC_50_) was determined.

***In vitro lipoxygenase (LOX) inhibitory activity***

In a 96-well plate, serial dilutions of the tested extract, and fractions (125- 0.98 µg/mL), as well as the isolated compounds and standards (250 - 0.98 µM) were prepared in DMSO. The LOX inhibitory activity was assessed colorimetrically based on the enzymatic oxidation of linoleic acid to the corresponding hydroperoxide as previously published method [7]. DMSO was used as solvent control instead of the tested sample. Zileuton was used as reference drug. The absorbances were recorded at 234nm. The % inhibition was calculated using the following formula:

LOX % Inhibition= [(100%IA−Inhibitor)/ 100% IA x 100]. Where IA: 100% initial activity.

The IC_50_ of each tested sample was determined.

**Cell culture**

The RAW264.7 macrophage cells were grown on DMEM media, supplemented with 10% FBS and 1% P/S (100 U/ml and 100 mg/ml, respectively). The RAW264.7 macrophage cells were kept in DMEM at 37 °C and 5% CO_2_. For the anti-inflammatory assays, cells were used at 60% confluency after a 24-hour acclimatization period.

***Cell viability assay***

The cell viability assays were performed using MTT reduction assay according to [8]. In a 96-well plate, the RAW264.7 macrophages were plated (1 × 10^16^ cells per well) for 24 h. After the incubation period, the culture medium was replaced by 200 µL serial dilutions of extract, fractions (0-1000 μg/ml), and isolated compounds (0-1000 µM) and further incubated for 24 h. The culture media were replaced by 90 µL of fresh culture medium. In each well, 5 mg/mL MTT in phosphate buffer (PBS, pH 7.4) was added and incubated for 5 h at 37 °C (5% CO_2_). The unreacted dye was removed and DMSO was added (200 µL/well) to dissolve the formazan crystals. The absorbances were recorded at 570 nm.

***Tested samples treatment***

Stock solutions of the extract, fractions (1 mg/mL) and the isolated compounds (1000 μM) were made by dissolving in 0.1% DMSO in PBS (pH 7.2). For experiments, they were further diluted with DMEM to obtain the desired concentration. In a 96-well plate, the RAW264.7 macrophages were seeded (4 × 10^3^ cells per well) overnight, then washed with DMEM. The macrophages were pre-treated with various concentrations of the extract, fractions (125- 0.98 µg/mL), isolated compounds, and standard drugs (125 - 0.06 µM) for 30 min, and further incubated with LPS (1 μg/mL) for 24 h [9]. For the negative control, cells were treated with 0.1% DMSO (negative control LPS-). After incubation, the monolayer of RAW 26.7 cells were washed with ice-cold PBS (pH 7.2), trypsinized, and treated with lysis buffer (1% Triton X-100, 50 mMTris–HCl, pH 7.4, 0.2% sodium dodecyl sulfate, 0.2% sodium deoxycholate, 1 mM phenylmethylsulfonyl fluoride, 1 mM sodium ethylenediaminetetraacetate, 5 μg/mL of leupeptin, 5 μg/mL of aprotinin) then centrifuged (5000 rpm for 10 min) to collect the supernatant, The cell lysates were stored at − 80 °C utilized for anti-inflammatory assays.

***iNOS inhibitory activity in RAW264.7 macrophage cells***

The inhibitory activity of Inducible nitric oxide synthase (iNOS) was determined in terms of nitric oxide (NO) concentration by measuring the quantity of nitrite in the cell culture supernatant using Griess reagent [10]. Equal volumes of each cell lysate (100 µL) and Griess reagent (1% sulfanilamide, 0.1% naphthylethylenediamine dihydrochloride, and 5% phosphoric acid) were mixed and incubated at room temperature for 10 min. Using a microplate reader, absorbances at 540 nm were measured. Parthenolide served as a positive control. The % inhibition was calculated using the following formula:

iNOS % Inhibition= [(OD control−OD sample)/ OD control x 100]. Where OD: optical denidensity concentrations at which the tested samples produced 50% inhibition of iNOS (IC_50_) were determined.

**Effect on** **cytokines secretion**

The concentration of the released cytokines (TNF-*α*, IL-1*β*, IL-6) were measured in the cell lysates using their respective ELISA kit according to the manufacturer’s instructions using BIOLINE ELISA READER.

**Identification and quantification of the silylated methylene chloride fraction (MCF) by GC-MS**

GC-MS of MCF was carried out according to [11] with slight modifications. Briefly, 100 mg of MCF were extracted with 5 mL 100% methanol with sonication for 30 min while shaking frequently. Debris was then removed using centrifugation at 12 000 g for 10 min. Three distinct samples were examined under the same circumstances. The extract was then divided into 100 mL screw-cap vials and allowed to evaporate under a stream of nitrogen gas until it was completely dry. The dried extract was combined with 150 mL of N-methyl-N-(trimethylsilyl)-triuoroacetamide (MSTFA), which had previously been diluted 1: 1% with anhydrous pyridine. This mixture was then incubated for 45 minutes at 60 C before being analyzed using GC-MS. On a Rtx5MS (30 m length, 0.25 mm inner diameter, and 0.25 mm lm), silylated derivative separation was accomplished.

**GC-MS metabolites identification.**

By mass matching to NIST, WILEY library database, previous literature [12, 13] and with standards when available, it was possible to identify both volatile and non-volatile silylated components. Prior to mass spectral matching, peaks were first deconvoluted using AMDIS software (www.amdis.net (accessed on 28 April 2023)).

**Statistical analysis**

All analyses were performed in triplicates, data were analyzed using Graph pad Prism 8® (San Diego, CA, USA), and data are represented as means (n=3) ± SD. One-way ANOVA was carried out to determine significant differences among means followed by Tukey’s multiple comparison test.

**Isolation of the major compounds from the methylene chloride fraction (MCF)**

Fraction II (3 g), eluted with 10% to 50% CHCl_3_ in *n*-hexane was chromatographed on a silica gel 60 column (30 L × 3 D cm) using *n*-hexane and gradually increasing the polarity by 1% ethyl acetate. The sub-fraction II-B (0.75 g) eluted with 3% ethyl acetate in *n*-hexane, showed a single spot that appeared violet when sprayed with *p*-anisaldehyde. The solvent was evaporated under reduced pressure and recrystallized with methanol to yield **compound A1 (**white powder, 98 mg). Fraction III (5 g), eluted with 60% to 80% CHCl_3_ in *n*-hexane was chromatographed on a silica gel 60 column (35 L × 4 L cm) using *n*-hexane and gradually increasing the polarity by 5% ethyl acetate. The sub-fraction III-A (0.85 g) eluted with 5% ethyl acetate in *n*-hexane, showed a single spot that appeared violet when sprayed with *p*-anisaldehyde. The solvent was evaporated under reduced pressure and recrystallized with methanol to yield **compound A2 (**white needles, 100 mg). Fraction V (4.25g), eluted with 20% to 30% ethyl acetate in methylene chloride was chromatographed on a silica gel 60 column (35 L × 4 D cm) adopting isocratic elution using *n*-hexane and ethyl acetate (85:15 v/v). **Sub Fr. V-B** (0.9 g) was purified on a silica gel 60 column (25 L ҳ 1 D cm), by gradient elution using *n*-hexane and gradually increasing the polarity by 5% ethyl acetate to give **compound A3** (white needles, 110 mg) at system of 20% ethyl acetate in *n*-hexane. Fraction VI (4.1 g), eluted with 40% ethyl acetate in methylene chloride, was loaded on was loaded onto a silica gel 60 column (35 L X 4 D cm) adopting gradient elution using *n*-hexane and gradually increasing the polarity by 5% ethyl acetate. Sub-Fraction VI-C (1 g)**,** eluted using 20% ethyl acetate in *n*-hexane, was purified on a silica gel RP-18 column (20 L ҳ 2 D cm). Gradient elution was carried out using methanol-water mixtures of increasing polarity, to obtain **compound A4** (white needles, 115 mg).

***Data of the isolated compounds (A1-A4)***

**Compound A1:** White powder; melting point: 61-63 °C. ^1^H-NMR (400 MHz, CDCl_3_): δ (ppm); 0.8612 (3H, t, *J*= 6.8 Hz, H-18), 1.2512-1.3018 (20H, m, H_2_-4 to H_2_-7, H_2_-12 to H_2_-17), 1.6214 (2H, m, H-3), 1.9901 (4H, m, H_2_-8 and H_2_-11), 2.2121 (2H, t, *J*= 7.6; H-2), 5.3345 (2H, m, H-9 and H-10), 5.5213 (1H, br s, N-H); 5.8721(1H, br s, N-H). ^13^C-NMR (100 MHz, CDCl_3_): δ (ppm); 175.97, 130.02, 129.78 (C-9, C-10), 35.99, 31.9-, 29.75, 29.68, 29.51, 29.31, 29.23, 29.18, 29.10, 28.83, 27.19, 27.14, 25.51 (13CH_2_), 22.68 (C-17), 14.12 (C-18).

**Compound A2:** White needles; melting point: 175-177 °C. ^1^H-NMR (400 MHz, CDCl_3_): δ (ppm); 0.6527 (3H, s, Me-18), 0.7627 (3H, d*, J =* 8.5 Hz, Me-27), 0.8871 (3H, t, *J* = 6.5 Hz, Me-29), 0.7289 (3H, d, *J =* 6.7 Hz, Me-26), 0.9571 (3H, d, *J* = 7.5 Hz, Me-21), 0.9795 (3H, s, Me-19), 3.1911 (1H, m, H-3), 5.3812 (1H, bs, H-6), 5.0586 (1H, dd, *J* = 8.5, 15.15 Hz, H-22), 4.9044 (1H, dd, *J* = 8.6, 15.12 Hz, H-23). ^13^C-NMR (100 MHz, CDCl_3_): δ (ppm); 37.23 (C-1), 32.34 (C-2), 71.27 (C-3), 40.48 (C-4), 140.80 (C-5), 121.49 (C-6), 31.85 (C7),31.15 (C-8), 51.22 (C-9), 36.45(C-10), 21.02(C-11),39.46 (C-12), 43.26 (C-13), 56.04 (C-14), 23.00 (C15), 28.19 (C-16), 55.91 (C-17),11.95 (C-18), 18.88 (C-19), 39.74 (C-20),21.14 (C-21), 138.29 (C22), 129.22 (C 23),49.46 (C-24), 29.10 (C-25), 19.26 (C-26),19.69 (C-27), 26.04 (C-28), 12.12 (C-29).

**Compound A3:** White needles; melting point: 95-97 °C. ^1^H-NMR (400 MHz, CDCl_3_): δ (ppm); 7.3357 (2H, t, *J*= 7Hz, H-3", H-5"), 7.2301 (1H, t, *J*= 7 Hz, H-4"), 7.2117 (2H, d, *J*= 7 Hz, H-2", H-6"), 7.1939 (1H, dd, *J*= 10, 15 Hz, H-3), 6.1161 (1H, dd, *J*= 10, 15 Hz, H-4), 6.0772 (1H, dt, *J*= 7, 15 Hz, H-5), 5.7464 (1H, d, *J*= 15 Hz, H-2), 5.3875 (1H, br s, NH), 3.6148 (2H, dt, *J*= 6, 7 Hz, H-2'), 2.8621 (2H, t, *J*= 7 Hz, H-3'), 2.1330 (2H, dt, *J*= 7, 7 Hz; H-6), 1.4157 (2H, tt , *J*= 7, 7 Hz, H-7) overlapping signals, 1.2817 (4H, m, H-8, H-9) overlapping signals, 0.8937 (3H, t, 7, H-10). ^13^C-NMR (100 MHz, CDCl_3_): δ (ppm); 167.26 (C-1), 123.23 (C-2), 136.30 (C-3), 126.30 (C-4), 140.56 (C-5), 28.23 (C-6), 29.57 (C-7), 31.75 (C-8), 22.57 (C-9), 13.88 (C-10), 40.83 (C-2'), 35.44 (C-3'), 139.73 (C-1''), 128.64 (C-2'', 6''), 128.43 (C-3'', 5''), 126.70 (C-4'').

**Compound A4:** White needles; melting point: 90-92 °C. ^1^H-NMR (400 MHz, CDCl_3_): δ (ppm); 0.8962 (3H, t, J = 6.9 Hz, H-10), 0.9307 (6H, d, J= 6.9 Hz, H-3’, H-4’), 1.3221 (4H, m, H-8 and H-9), 1.4202 (2H, m, H-7), 1.8062 (1H, m, H-2’ ), 2.0771 (2H, m, 6.9 Hz, H-6), 3.1379 (2H ,dd, J= 6.9, 6.1 Hz, H-1’), 5.5012 (1H, br s, N-H), 5.7845 (1H, d, J= 15 Hz, H-2), 6.0293 (1H, dt, J= 15, 6.9 Hz, H-5); 6.1348 (1H, dd, J= 15, 9.6 Hz, H-4); 7.1970 (1H, dd, J = 15, 9.6 Hz, H-3). ^13^C-NMR (100 MHz, CDCl_3_): δ (ppm); 167.77 (C-1), 142.83 (C-5), 140.93 (C-3), 128.41 (C-4), 121.65 (C-2), 47.27 (C-1'), 32.65 (C-6), 31.61 (C-8), 28.93 (C-2^'^), 28.66 (C-7), 22.37 (C-9), 20.35 (C-3^'^), 20.35 (C-4'), 14.02 (C-10).

***2.9.2. Identification of the isolated compounds (A1-A4)*** The ^1^H-NMR of **compound A1** showed a multiplet signal assigned to two olefinic methines at δ 5.3345. Multiplets assigned to methylenes of saturated carbon chains were observed at δ 1.2512-1.3018 and δ 1.6214. Proton signals of three allyl methylenes at δ 2.2121 (2H, t, *J*= 7.6 Hz), and 1.9901 (2H*2, each, m), a methyl signal [δ 0.8612 (3H, t, *J*=6.8 Hz] were detected. Two broad singlets were observed at δ 5.8721 and 5.5213 ppm each assigned to NH proton. The ^13^C-NMR spectrum of compound A3 displayed 18 carbon signals, one carbonyl (δ 175.97), two olefinic methines (δ 130.02, 129.78), fourteen methylenes (δ 35.99-22.68), and one methyl (δ 14.12). The results suggested that compound A3 was composed of 18 C fatty acids with one double bond. Furthermore, compound A3 was confirmed as an amide in which nitrogen was substituted for the acid part of the carboxyl group. Based on published data [14], **compound A1** could be identified as 9-cis-octadecenamide (oleamide). It is isolated for the first time from the genus *Anacyclus*.

In the 1H-NMR spectrum of **compound A2,** two singlets were detected at δ 0.6527 ppm (3H, Me-18) and 0.9795 ppm (3H, Me-19). Three doublets were observed at δ 0.9571 ppm (3H, *J*= 7.5 Hz, Me-21), δ 0.7289 ppm (3H, *J*= 6.7 Hz, Me-26), and δ 0.7627 ppm (3H, *J*= 8.5 Hz, Me-27). A triplet signal at δ 0.8871 ppm (3H, *J* = 6.5 Hz, Me-29). The proton corresponding to the H-3 was appeared as a multiplet at δ 3.49 ppm. Three olefinic protons were detected at δ 5.3812 (bs, H-6), δ 5.0586 (dd, 8.5, 15.15 Hz, H-22), δ 4.9044 (dd, 8.6, 15.12 Hz, H-23) justified a stigmasterol nucleus. In the ^13^C-NMR spectrum of **compound A2,** 29 carbon signals were detected confirming its steroidal nature; which were 6 methyls, 9 methylenes, 11 methines, and 3 quaternary carbons. olefinic carbons signals were observed at *δ* 140.80, 121.49, 138.29, and 129.22 ppm corresponding to C-5, C-6, C-22, and C-23, respectively. The carbon signal of C-3 resonated at *δ* 71.27 ppm. With respect to the previous discussions and published data [15], **compound A2** could be identified as stigmasterol.

The ^1^H NMR spectrum of compound A3 detected a phenyethylamine moiety characterized by 5 aromatic protons at δ 7.3357 (2H, t, *J* = 7 Hz, H-3″and H-5″), 7.2301(1H, t, *J* = 7Hz, H-4″), 7.2117 (2H, d, *J* = 7 Hz, H-2″ and H-6) and 2 methylenes at δ 3.6148 (2H,dt, *J* = 6, 7 Hz, H2-2′), 2.8621 (2H, t, J = 7 Hz, H2-3) in an AA'BB'’ system. The presence of two double bonds was determined by the olefinic protons signals at δ 7.1939 (1H, dd, *J* = 10, 15 Hz, H-3), 6.1161 (1H, dd, *J* = 10, 15 Hz, H-4), 6.0772 (1H, dt, J= 7, 15 Hz, H-5), 5.7464 (1H, d, J = 15 Hz, H-2). Four methylenes were observed at δ 2.1330 (2H, dt, *J* = 7, 7 Hz, H-6), 1.4157 (2H, tt, *J* = 7,7 Hz, H-7), and 1.2817 (4H, m, H-8 and H-9). A signal of a methyl group was detected at δ 0.8937 (3H, t, *J* = 7 Hz, H-10). A broad singlet at δ 5.3875 was assigned for the amide proton. The ^13^C-NMR data showed sixteen signals corresponding to one amide carbonyl carbon (δ 167.26), one benzene ring (δC 139.73, 128.64, 128.43, 126.7), four olefins (δC 140.56, 136.30, 126.30, 123.23), six methylenes (δC 40.83, 35.44, 31.75, 29.57, 28.23, 22.57), and a methyl group (δ C 13.88). **Compound A3** could be identified as 2E,4E-deca-2,4-dienoic acid 2-phenylethyl amide [16]**.**

The ^1^H NMR spectrum of **compound A4** revealed characteristic signals for an *N*-iso­butylamide group at δ 3.1379 ppm (2H, dd, *J*= 6.9, 6.1 Hz, H-1’), δ 1.8062 ppm (1H, m, H-2’) and δ 0.9307 ppm (6H, d, *J*= 6.9 Hz, H-3’, H-4’), together with a broad singlet of amide proton at δ 5.5012 ppm (1H). Four olefinic methines signals were observed at δ 5.7845 (1H, d, *J*= 15 Hz, H-2), 6.0293 (1H, dt, *J*= 15, 6.9 Hz, H-5), 6.1348 (1H, dd, *J*= 15, 9.6 Hz, H-4) and 7.1970 1H, dd, *J* = 15, 9.6 Hz, H-3) ppm. Multiplet signals at δ 1.3221 (4H, m, H-8 and H-9, overlapping signals) (6H) and 1.4202 (2H, m, H-7) and 2.0771 (2H, m, H-6) were assigned for the geminal protons (H-8, H-9), H-7) and H6, respectively. One methyl proton (H-10) was observed at δ 0.8962 (3H, t, *J* = 6.9 Hz). The ^13^C-NMR of **compound A4** displayed 14 carbon signals with a characteristic signal of the amide carbonyl carbon δ 167.77 (C-1). Based on the previous discussion and published data, **compound A4** was identified as deca-2E,4E-dienoic acid isobutylamide (pellitorine) [17].

**Molecular docking**

All the molecular docking studies were achieved using Molecular Operating Environment (MOE, 2019.0102) software. The X-ray crystal structure of the Acetylcholinesterase Enzyme (PDB code: 4EY7), in complex with Donepezil and of COX-2 co-crystallized with Celecoxib as inhibitor (PDB code: 3LN1), was imported from the protein data bank. Regarding AChE protein structure, it was prepared for docking by removal of water molecules except for those involved in water-mediated hydrogen bonds with Donepezil. As for COX-2 protein structure, the enzyme was prepared by the removal of all ligands and water molecules that are not involved in the binding. The protein structures were prepared for docking study using Protonate 3D protocol in MOE with default options. The structures of the compounds were constructed using ChemBioDraw Ultra 17.0 and their SMILES were copied to MOE. 3D Protonation of the compounds was carried out using the precise mode in the “Protonate” tool and the most prevalent ionized form was selected for subsequent steps. Energy minimization of the structures using Amber10:EHT forcefield and a gradient of 0.05 was applied using “Energy Minimize” tool. The partial charges were automatically calculated for each molecule. Conformational analysis was run using the default settings for systematic search. The least energy conformer of each molecule was saved to another database to be docked into the binding site of the target enzymes. The Triangle Matcher placement method and London dG scoring function were used for the evaluation of the binding pattern and binding affinity of the ligands. The co-crystalized ligands were used to define the active site for docking. The docking protocol was first validated by redocking the co-crystallized ligands in the active site of the enzymes with energy score (S) = -9.1833 kcal/mol and root mean square deviation (RMSD) of 0.1402 Å for AChE and energy score (S) = -9.6345 kcal/mol and root mean square deviation (RMSD) of 0.0903 Å for COX-2. The validated docking protocol was then used to study the ligand-target interactions for the identified compounds in the active site of the target enzymes. This was carried out to explore their binding modes to justify the promising inhibitory activity.

**Molecular dynamic (MD) simulations**

***System preparation***

The crystal structure of the human acetylcholinesterase receptor (AChE), and cyclooxygenase-2 (COX-2) receptor was retrieved from the protein data bank with codes 4EY7 [18] , 3LN1 [19], respectively. These structures were then prepared for molecular dynamics (MD) studies using UCSF Chimera [20]. Using PROPKA pH was fixed and optimized to 7.5 [21]. The structures were drawn using ChemBioDraw Ultra 12.1[22]. Altogether, all four prepared systems were subjected to 60 ns MD simulations as described in the simulation section.

The integration of Molecular dynamic (MD) simulations in biological systems' study enable exploring the physical motion of atoms and molecules that cannot be easily accessed by any other means [23]. The insight extracted from performing this simulation provides an intricate perspective into the biological systems' dynamical evolution, such as conformational changes and molecule association [23]. The MD simulations of all systems were performed using the GPU version of the PMEMD engine present in the AMBER 18 package [24].

The partial atomic charge of each compound was calculated with ANTECHAMBER's General Amber Force Field (GAFF) technique [25]. The Leap module of the AMBER 18 package implicitly solvated each system within an orthorhombic box of TIP3P water molecules within 10 Å of any box edge. The Leap module was used to neutralize each system by incorporating Na^+^ and Cl^-^ counter ions. A 2000-step initial minimization of each system was carried out in the presence of a 500 kcal/mol applied restraint potential, followed by a 1000-step full minimization using the conjugate gradient algorithm without restraints.

During the MD simulation, each system was gradually heated from 0K to 300K over 500ps, ensuring that all systems had the same number of atoms and volume. The system's solutes were subjected to a 10kcal/mol potential harmonic constraint and a 1ps collision frequency. Following that, each system was heated and equilibrated for 500ps at a constant temperature of 300K.To simulate an isobaric-isothermal (NPT) ensemble, the number of atoms and pressure within each system for each production simulation were kept constant, with the system's pressure maintained at 1 bar using the Berendsen barostat [26].

For 60 ns, each system was MD simulated. The SHAKE method was used to constrain the hydrogen bond atoms in each simulation. Each simulation used a 2fs step size and integrated an SPFP precision model. An isobaric-isothermal ensemble (NPT) with randomized seeding, constant pressure of 1 bar, a pressure-coupling constant of 2ps, a temperature of 300K, and a Langevin thermostat with a collision frequency of 1ps was used in the simulations.

**Post-MD Analysis**

After saving the trajectories obtained by MD simulations every 1 ps, the trajectories were analyzed using the AMBER18 suite's CPPTRAJ module [27]. The Origin [28] data analysis program and Chimera [20] were used to create all graphs and visualizations.

***In silico* ADME profile *and BBB permeability prediction***

The Swiss ADME web server (<http://www.swissadme.ch/index.php>) was utilized to predict the drug-likeness profiles and pharmacokinetic properties of the examined compounds where the SMILES of the compounds were directly inserted into the webpage then the prediction procedure was achieved [29]. Moreover, the free accessible web server pkCSM (<http://biosig.unimelb.edu.au/pkcsm/prediction>) was used to predict BBB permeability of the assessed compounds [30].

**Supplementary figures**

| 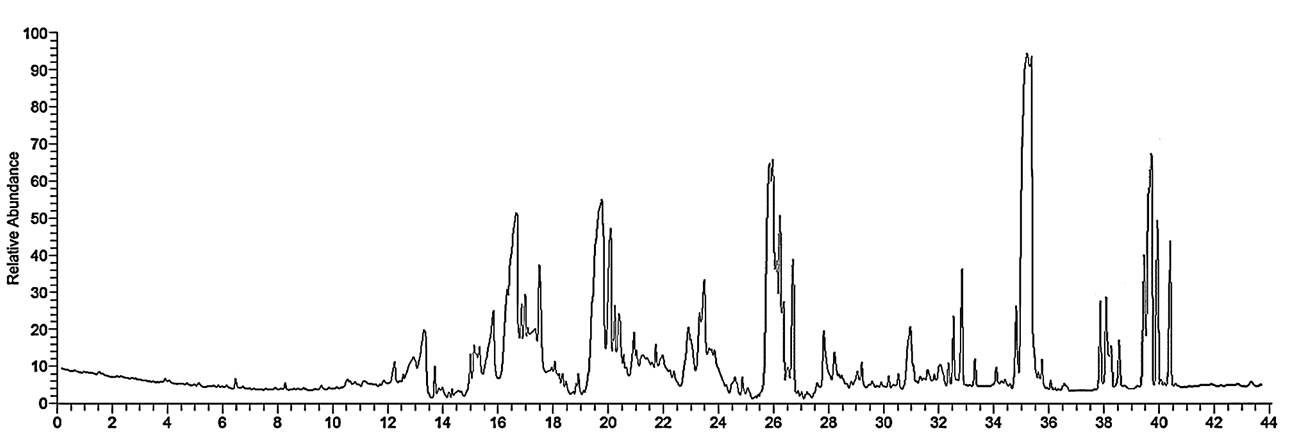 |
| --- |

**Fig. S1:** Gas chromatography coupled with mass-spectrometry (GC-MS) total ion chromatograms (TIC) of silylated compounds in the methylene chloride fraction *Anacyclus pyrethrum* roots

| 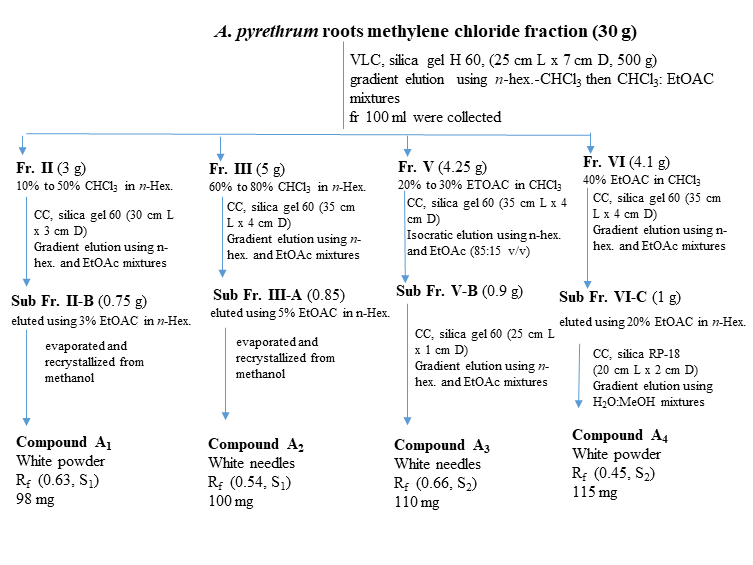 |
| --- |

**Fig. S2** Scheme for chromatographic fractionation of *A. pyrethrum* roots methylene chloride fraction

A1: oleamide; A2: stigmasterol; A3: deca-2E,4E-dienoic acid 2-phenylethylamide; A4: pellitorine EtOAc; ethyl acetate; Fr.: fraction; MeOH: methanol; *n*-Hex.: *n*-hexane; S_1_: *n*-hexane-ethyl acetate (80:20 *v*/*v*); S_2_: methylene chloride-methanol (97:3 *v*/*v*); Sub Fr.: subfraction.

| 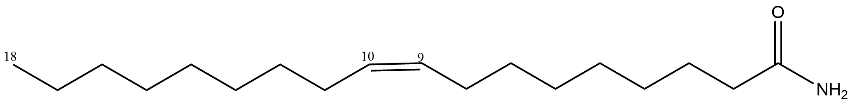**A1** | 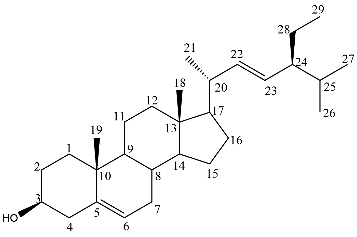 |
| --- | --- |
|  | **A2** |
| **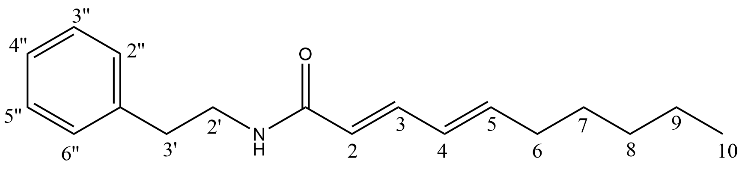** | **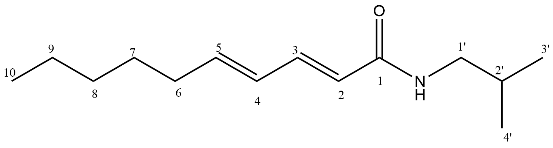** |
| **A3** | **A4** |

**Fig. S3** Structure of the isolated compounds**, A1**: oleamide; **A2**: stigmasterol; **A3**: deca-2E,4E-dienoic acid 2-phenylethylamide; **A4**: pellitorine

|  |  |
| --- | --- |

**Fig. S4** Line graph representing the Effect of A) ME, MCF and BF, (B) the four isolated compounds (A1-A4) on the RAW264.7 macrophages viability using the MTT assay.

**Fig. S5** Microscopic observation of the effect (A) ME, (B) MCF, (C) BF at different concentrations (4-1000 μg/mL), (D) A1, (E) A2, (F) A3, (G) A4 at different concentrations (4-1000 μM) on RAW264.7 macrophages at 24 hours against DMSO control


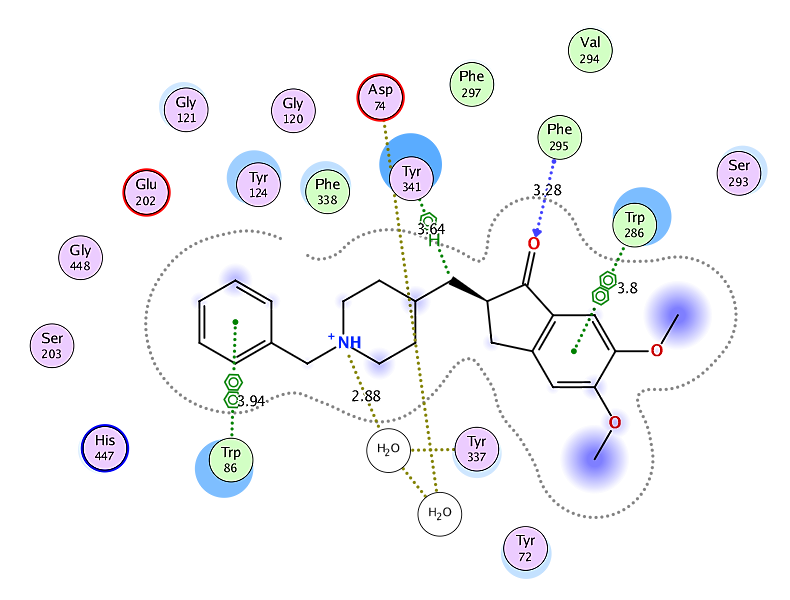


**Fig. S6** 2D interaction diagram showing Donepezil interactions with the key amino acids in AChE active site.


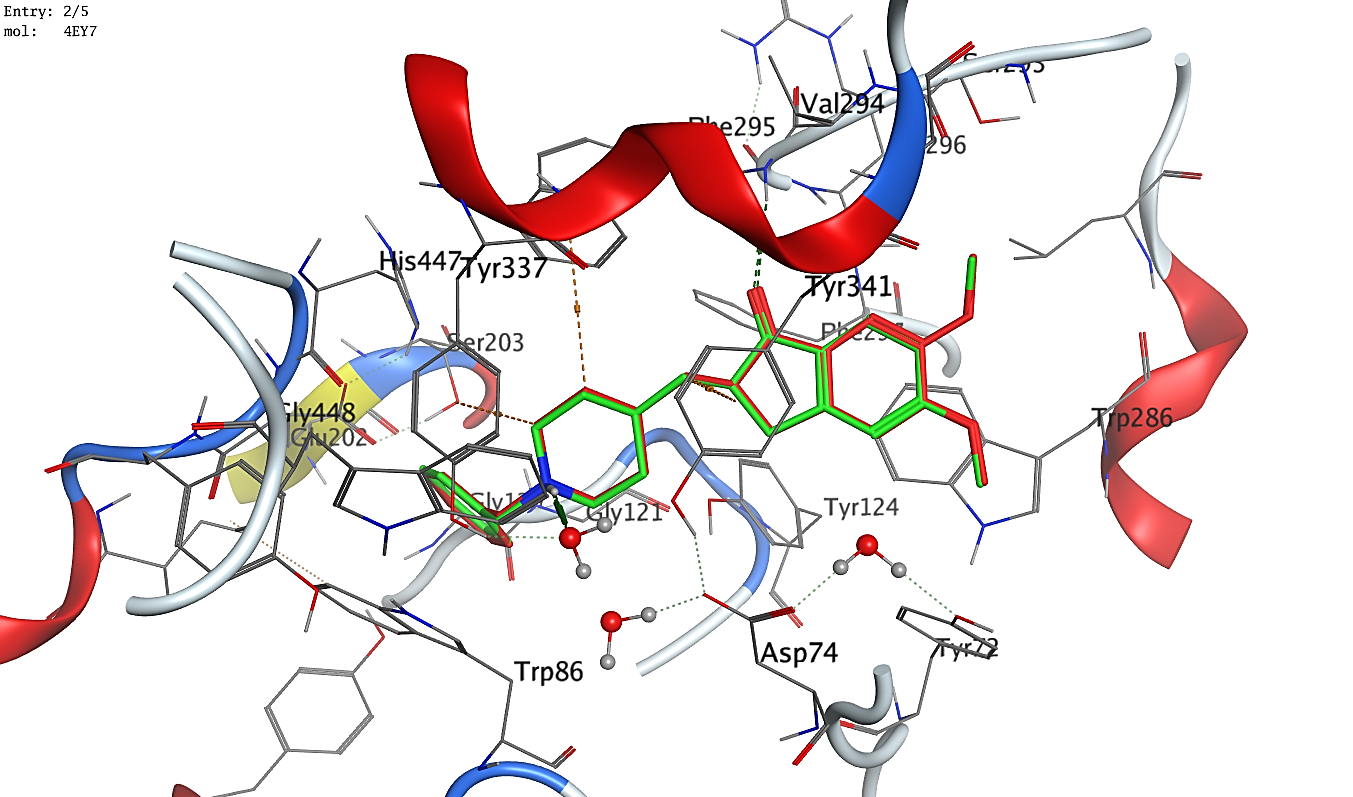


**Fig. S7** 3D representation of the superimposition of the docked pose (red) and the co-crystallized ligand Donepezil (green) in the AChE binding site (RMSD = 0.1402 Å).


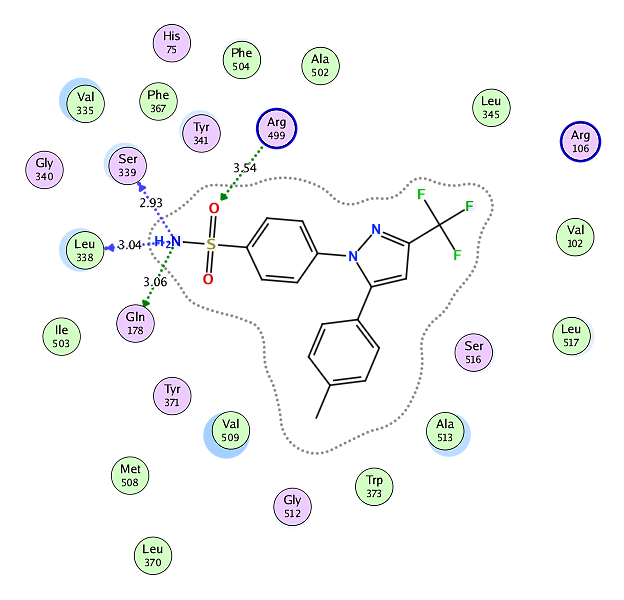


**Fig. S8** 2D interaction diagram showing Celecoxib interactions with the key amino acids in COX-2 active site.


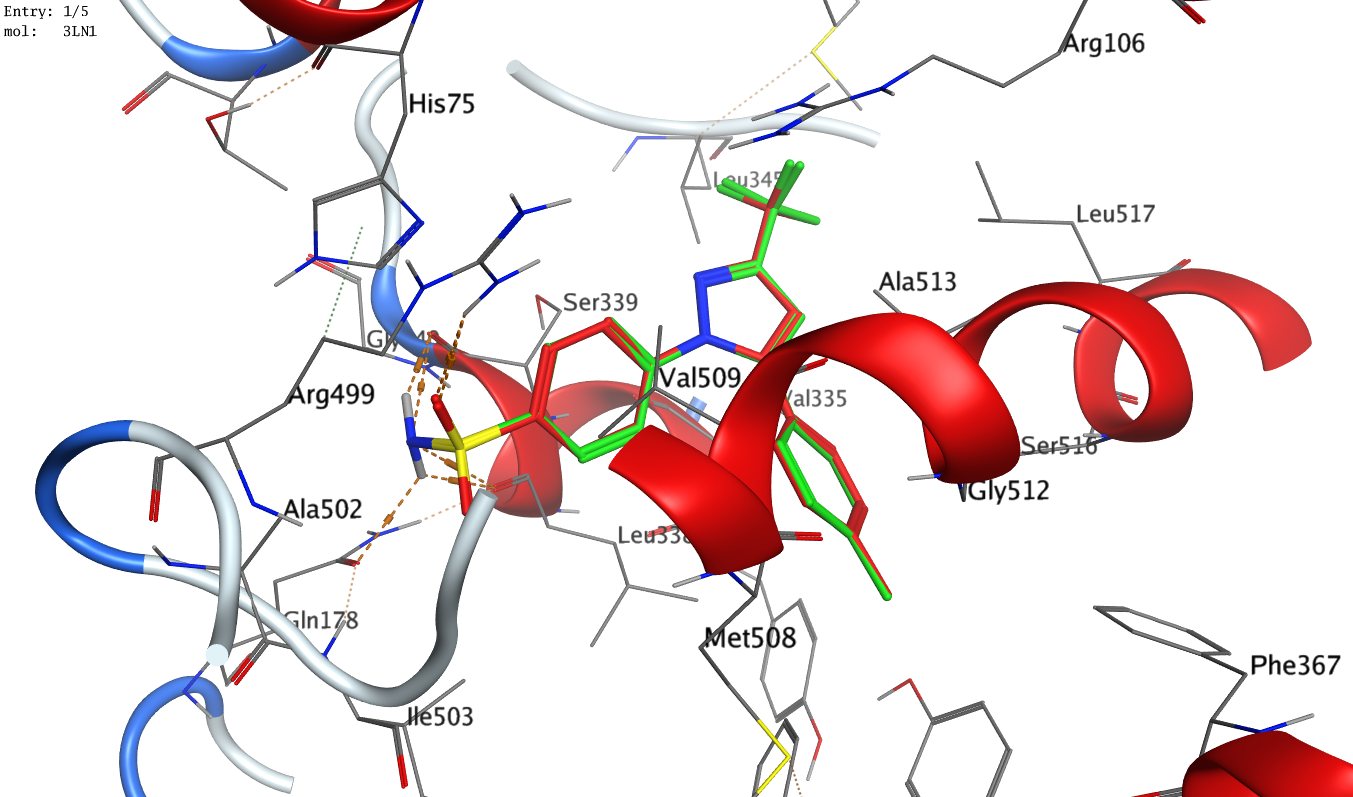


**Fig. S9** 3D representation of the superimposition of the docked pose (red) and the co-crystallized ligand Celecoxib (green) in the COX-2 binding site (RMSD = 0.0903 Å).


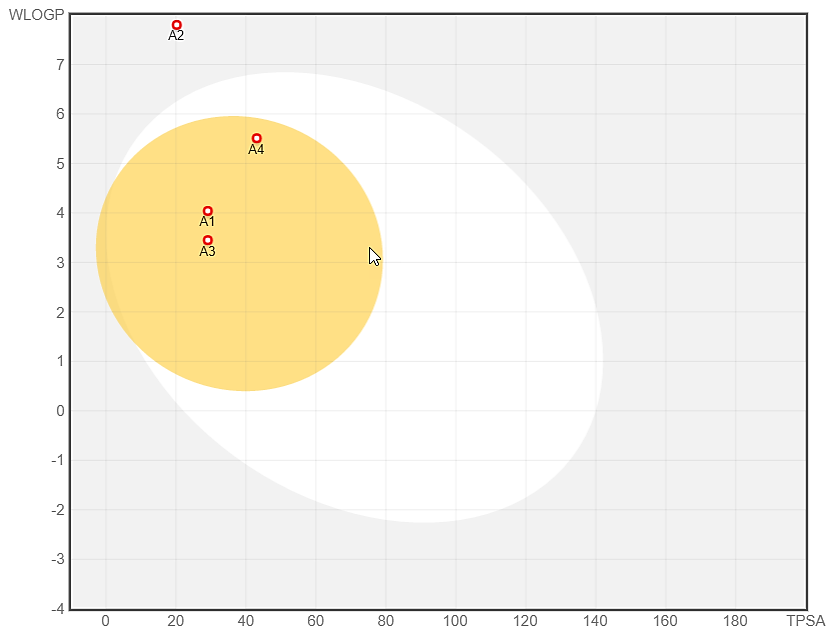


**Fig. S10** The boiled egg chart of the isolated compounds

**References**

1. Argoti JC, Linares‐Palomino PJ, Salido S et al. On‐line activity screening for radical scavengers from *Baccharis chilco*. Chem Biodivers. 2013; 10: 189-197. DOI: https://doi.org/10.1002/cbdv.201200192

2. Faitanin RD, Gomes JV, Rodrigues PM et al. Chemical study and evaluation of antioxidant activity and α-glucosidase inhibition of Myrciaria strigipes O. Berg (Myrtaceae). J Appl Pharm Sci. 2018; 8: 120-125. DOI: https://doi.org/10.7324/JAPS.2018.8317

3. Justino AB, Miranda NC, Franco RR et al. Annona muricata Linn. leaf as a source of antioxidant compounds with in vitro antidiabetic and inhibitory potential against α-amylase, α-glucosidase, lipase, non-enzymatic glycation and lipid peroxidation. Biomed Pharmacother. 2018; 100: 83-92. DOI: https://doi.org/10.1016/j.biopha.2018.01.172

4. Santos JS, Brizola VRA, Granato D. High-throughput assay comparison and standardization for metal chelating capacity screening: A proposal and application. Food Chem. 2017; 214: 515-522. DOI: https://doi.org/10.1016/j.foodchem.2016.07.091

5. Kia Y, Osman H, Kumar RS et al. Ionic liquid mediated synthesis of mono-and bis-spirooxindole-hexahydropyrrolidines as cholinesterase inhibitors and their molecular docking studies. Bioorg Med Chem. 2014; 22: 1318-1328. DOI: https://doi.org/10.1016/j.bmc.2014.01.002

6. George A, Chinnappan S, Chintamaneni M et al. Anti-inflammatory effects of Polygonum minus (Huds) extract (Lineminus™) in in-vitro enzyme assays and carrageenan induced paw edema. BMC Complement Altern Med. 2014; 14: 1-7. DOI: https://doi.org/10.1186/1472-6882-14-355

7. Costamagna MS, Zampini IC, Alberto MR et al. Polyphenols rich fraction from Geoffroea decorticans fruits flour affects key enzymes involved in metabolic syndrome, oxidative stress and inflammatory process. Food Chem. 2016; 190: 392-402. DOI: https://doi.org/10.1016/j.foodchem.2015.05.068

8. Manosroi A, Saraphanchotiwitthaya A, Manosroi J. Immunomodulatory activities of Clausena excavata Burm. f. wood extracts. J Ethnopharmacol. 2003; 89: 155-160. DOI: https://doi.org/10.1016/s0378-8741(03)00278-2

9. Jeong HY, Sung G-H, Kim JH et al. Syk and Src are major pharmacological targets of a Cerbera manghas methanol extract with kaempferol-based anti-inflammatory activity. J Ethnopharmacol. 2014; 151: 960-969. DOI: https://doi.org/10.1016/j.jep.2013.12.009

10. Stone WL, Yang H, Qui M. Assays for nitric oxide expression. Mast Cells: Methods and Protocols. 2005; 245-256.

11. Farag MA, Khattab AR, Shamma S et al. Profiling of primary metabolites and volatile determinants in mahlab cherry (Prunus mahaleb L.) seeds in the context of its different varieties and roasting as analyzed using chemometric tools. Foods. 2021; 10: 728. DOI: https://doi.org/10.3390/foods10040728

12. Jawhari FZ, El Moussaoui A, Bourhia M et al. Anacyclus pyrethrum (L): Chemical composition, analgesic, anti-inflammatory, and wound healing properties. Molecules. 2020; 25: 5469. DOI: https://doi.org/10.3390/molecules25225469

13. Veryser L, Taevernier L, Wynendaele E et al. N-alkylamide profiling of Achillea ptarmica and Achillea millefolium extracts by liquid and gas chromatography–mass spectrometry. J Pharm Anal. 2017; 7: 34-47. DOI: https://doi.org/10.1016/j.jpha.2016.09.005

14. Ingolfsdottir K, Gissurarson S, Nenninger A et al. Biologically active alkamide from the lichen Stereocaulon alpinum. Phytomedicine. 1997; 4: 331-334. DOI: https://doi.org/10.1016/S0944-7113(97)80042-6

15. Chaturvedula VSP, Prakash I. Isolation of Stigmasterol and?-Sitosterol from the dichloromethane extract of Rubus suavissimus. Int Curr Pharm J. 2012; 1: 239-242. DOI: https://doi.org/10.3329/icpj.v1i9.11613

16. Althaus JB, Malyszek C, Kaiser M et al. Alkamides from Anacyclus pyrethrum L. and their in vitro antiprotozoal activity. Molecules. 2017; 22: 796. DOI: https://doi.org/10.3390/molecules22050796

17. Hamimed S, Boulebda N, Laouer H et al. Bioactivity-guided isolation of alkamides from a cytotoxic fraction of the ethyl acetate extract of Anacyclus pyrethrum (L.) DC. roots. Curr Issues Pharm Med Sci. 2018; 31: 180-185. DOI: https://doi.org/10.1515/cipms-2018-0033

18. Cheung J, Rudolph MJ, Burshteyn F et al. Structures of human acetylcholinesterase in complex with pharmacologically important ligands. J Med Chem. 2012; 55: 10282-10286. DOI: https://doi.org/10.1021/jm300871x

19. Wang JL, Limburg D, Graneto MJ et al. The novel benzopyran class of selective cyclooxygenase-2 inhibitors. Part 2: The second clinical candidate having a shorter and favorable human half-life. Bioorganic & medicinal chemistry letters. 2010; 20: 7159-7163. DOI: https://doi.org/10.1016/j.bmcl.2010.07.054

20. Pettersen EF, Goddard TD, Huang CC et al. UCSF Chimera—a visualization system for exploratory research and analysis. J Comput Chem. 2004; 25: 1605-1612. DOI: https://doi.org/10.1002/jcc.20084

21. Li H, Robertson AD, Jensen JH. Very fast empirical prediction and rationalization of protein pKa values. Proteins: Structure, Function, and Bioinformatics. 2005; 61: 704-721. DOI: https://doi.org/10.1002/prot.20660

22. Halford B. Reflections on CHEMDRAW. Chem, Eng News. 2014; 92: 26-27.

23. Hospital A, Goñi JR, Orozco M et al. Molecular dynamics simulations: advances and applications. Advances and Applications in Bioinformatics and Chemistry. 2015; 37-47. DOI: https://doi.org/10.2147/AABC.S70333

24. Lee T-S, Cerutti DS, Mermelstein D et al. GPU-accelerated molecular dynamics and free energy methods in Amber18: performance enhancements and new features. J Chem Inf Model. 2018; 58: 2043-2050. DOI: https://doi.org/10.1021/acs.jcim.8b00462

25. Wang J, Wang W, Kollman PA et al. Automatic atom type and bond type perception in molecular mechanical calculations. Journal of molecular graphics and modelling. 2006; 25: 247-260. DOI: https://doi.org/10.1016/j.jmgm.2005.12.005

26. Berendsen HJ, Postma Jv, Van Gunsteren WF et al. Molecular dynamics with coupling to an external bath. The Journal of chemical physics. 1984; 81: 3684-3690. DOI: https://doi.org/10.1063/1.448118

27. Roe DR, Cheatham III TE. PTRAJ and CPPTRAJ: software for processing and analysis of molecular dynamics trajectory data. Journal of chemical theory and computation. 2013; 9: 3084-3095. DOI: https://doi.org/10.1021/ct400341p

28. Seifert E. OriginPro 9.1: scientific data analysis and graphing software-software review. J Chem Inf Model. 2014; 54: 1552. DOI: https://doi.org/10.1021/ci500161d

29. Daina A, Michielin O, Zoete V. SwissADME: A free web tool to evaluate pharmacokinetics, drug-likeness and medicinal chemistry friendliness of small molecules. Sci Rep. 2017; 7. DOI: 10.1038/srep42717

30. Pires DE, Blundell TL, Ascher DB. pkCSM: predicting small-molecule pharmacokinetic and toxicity properties using graph-based signatures. J Med Chem. 2015; 58: 4066-4072. DOI: https://doi.org/10.1021/acs.jmedchem.5b00104
